# Factors associated with anxiety disorder comorbidity

**DOI:** 10.1016/j.jad.2022.11.051

**Published:** 2023-02-15

**Authors:** Molly R. Davies, Kiran Glen, Jessica Mundy, Abigail R. ter Kuile, Brett N. Adey, Chérie Armour, Elham Assary, Jonathan R.I. Coleman, Kimberley A. Goldsmith, Colette R. Hirsch, Matthew Hotopf, Christopher Hübel, Ian R. Jones, Gursharan Kalsi, Georgina Krebs, Andrew M. McIntosh, Geneviève Morneau-Vaillancourt, Alicia J. Peel, Kirstin L. Purves, Sang Hyuck Lee, Megan Skelton, Daniel J. Smith, David Veale, James T.R. Walters, Katherine S. Young, Johan Zvrskovec, Gerome Breen, Thalia C. Eley

**Affiliations:** a Institute of Psychiatry, Psychology and Neuroscience, King's College London, Denmark Hill, Camberwell, London, UK; b National Institute for Health and Care Research (NIHR) Biomedical Research Centre, South London and Maudsley Hospital, London, UK; c Stress, Trauma & Related Conditions (STARC) research lab, School of Psychology, Queen's University Belfast (QUB), Belfast, UK; d South London and Maudsley NHS Foundation Trust, Denmark Hill, Camberwell, London, UK; e Department of Medical Epidemiology and Biostatistics, Karolinska Institutet, Stockholm, Sweden; f National Centre for Register-based Research, Aarhus Business and Social Sciences, Aarhus University, Aarhus, Denmark; g National Centre for Mental Health, Division of Psychiatry and Clinical Neuroscience, Cardiff University, Cardiff, UK; h Research Department of Clinical, Educational and Health Psychology, University College London, 1-19 Torrington Place, London, UK; i Division of Psychiatry, Centre for Clinical Brain Sciences, University of Edinburgh, Edinburgh, UK

**Keywords:** Depressive disorders, Anxiety disorders, Comorbidity, Polygenic risk score, Affective disorders

## Abstract

**Background:**

Anxiety and depressive disorders often co-occur and the order of their emergence may be associated with different clinical outcomes. However, minimal research has been conducted on anxiety-anxiety comorbidity. This study examined factors associated with anxiety comorbidity and anxiety-MDD temporal sequence.

**Methods:**

Online, self-report data were collected from the UK-based GLAD and COPING NBR cohorts (*N* = 38,775). Logistic regression analyses compared differences in sociodemographic, trauma, and clinical factors between single anxiety, anxiety-anxiety comorbidity, anxiety-MDD (major depressive disorder) comorbidity, and MDD-only. Additionally, anxiety-first and MDD-first anxiety-MDD were compared. Differences in familial risk were assessed in those participants with self-reported family history or genotype data.

**Results:**

Anxiety-anxiety and anxiety-MDD had higher rates of self-reported anxiety or depressive disorder diagnoses, younger age of onset, and higher recurrence than single anxiety. Anxiety-MDD displayed greater clinical severity/complexity than MDD only. Anxiety-anxiety had more severe current anxiety symptoms, less severe current depressive symptoms, and reduced likelihood of self-reporting an anxiety/depressive disorder diagnosis than anxiety-MDD. Anxiety-first anxiety-MDD had a younger age of onset, more severe anxiety symptoms, and less likelihood of self-reporting a diagnosis than MDD-first. Minimal differences in familial risk were found.

**Limitations:**

Self-report, retrospective measures may introduce recall bias. The familial risk analyses were likely underpowered.

**Conclusions:**

Anxiety-anxiety comorbidity displayed a similarly severe and complex profile of symptoms as anxiety-MDD but distinct features. For anxiety-MDD, first-onset anxiety had an earlier age of onset and greater severity than MDD-first. Anxiety disorders and comorbidity warrant further investigation and attention in research and practice.

## Highlights

• Minimal research has been conducted on anxiety-anxiety comorbidity.
• Anxiety-anxiety displayed similarly severe/complex profiles of symptoms as anxiety-MDD.
• Anxiety-anxiety had higher current anxiety but lower current depressive symptom severity than anxiety-MDD.
• Anxiety-MDD first-onset anxiety had a younger age of onset and more severe symptoms.
• Lower self-reported diagnosis rates may indicate less recognition of anxiety than MDD.

## 1 Background

Anxiety and depressive disorders are the most common mental health disorders worldwide (Bandelow and Michaelis, 2015; Kessler et al., 2005) and cause significant distress and impairment. Together, they account for 10 % of years lived with disability worldwide (World Health Organization, 2017). The substantial burden of these disorders is in part due to their early onset and chronicity (Costello et al., 2005). Comorbidity over the lifespan is so common as to be the rule rather than the exception (e.g., Brown et al., 2001; Lamers et al., 2011). Specifically, findings indicate as many as 63–67 % of people experience concurrent anxiety and depressive comorbidity, whilst 75–81 % experience comorbidity across the lifespan (Lamers et al., 2011). Similarly, 51.7 % of major depressive disorder (MDD) cases have a lifetime anxiety disorder (Kessler et al., 2015).

Prior research has mainly focussed on anxiety-depression comorbidity. Compared to those with a single diagnosis of anxiety or depressive disorder, individuals with comorbid anxiety-depression report: higher rates of trauma, more functional impairment, greater psychiatric symptom severity, increased suicide ideation or attempts, earlier onset, and more chronic or recurrent symptoms (Cyranowski et al., 2012; Kessler et al., 2015; Lamers et al., 2011; Moffitt et al., 2007; Ruscio et al., 2017). As such, comorbidity may confer a greater burden on individuals and thus be critical for understanding clinical outcomes. Furthermore, the temporal sequence of the onset of comorbid disorders may be clinically relevant. Notably, anxiety disorders often precede depressive disorders (Brown et al., 2001; de Graaf et al., 2003; Kessler et al., 2015; Lamers et al., 2011; Solmi et al., 2022). In cases of anxiety-depression comorbidity, individuals with preceding anxiety display more chronic and severe anxiety symptoms and an earlier age of onset than those with preceding depression (Lamers et al., 2011). The onset of an anxiety disorder therefore reflects risk for subsequent depressive disorders.

Minimal research has been conducted on comorbidity between the individual anxiety disorders. We are aware of one prior study on a sample with a current anxiety disorder which investigated differences in factors associated with anxiety-anxiety comorbidity compared to single anxiety and anxiety-depression comorbidity, using data from the Netherlands Study of Depression and Anxiety (NESDA; Hofmeijer-Sevink et al., 2012). Anxiety-anxiety comorbidity was associated with greater symptom severity and disability than single anxiety, but less severe depressive symptoms, lower disability, and lower rates of treatment receipt than anxiety-depressive comorbidity. However, individuals with anxiety-anxiety comorbidity also exhibited an earlier age of onset and more chronic course than those with either single anxiety or anxiety-depression comorbidity. These findings demonstrated that anxiety-anxiety comorbidity status is clinically important, representing a more complex presentation than single anxiety and longer duration than anxiety-depression comorbidity (Hofmeijer-Sevink et al., 2012).

Notably, in the NESDA study, those with anxiety-anxiety comorbidity more frequently reported having a family history of anxiety and/or depressive disorders than the single anxiety and anxiety-MDD groups (Hofmeijer-Sevink et al., 2012). This raises the possibility that individuals with anxiety-anxiety comorbidity have a higher genetic risk for anxiety disorders. However, genetic factors have remained largely unexplored in this context, despite twin studies demonstrating that genetics account for an estimated 30–50 % of risk for individual anxiety disorders (e.g., Hettema et al., 2001; Scaini et al., 2014; Smoller, 2016; Sullivan et al., 2000; Tambs et al., 2009). Recent advances in genetics enable the calculation of polygenic risk scores (PRS). These composite scores represent part of an individual's genetic risk for a disorder and can be used to compare differences in genetic risk between groups (International Schizophrenia Consortium et al., 2009; Lewis and Vassos, 2020; Wray et al., 2007). At present, a polygenic score for anxiety disorders explains 0.5 % of the variance in case-control status (Purves et al., 2020). Anxiety disorders have also been associated with higher polygenic risk for attention deficit hyperactivity disorder (ADHD; Du Rietz et al., 2018), schizophrenia (Crouse et al., 2021; Richards et al., 2019; Zheutlin et al., 2019), autism spectrum disorder (ASD; Wendt et al., 2020), MDD (Demirkan et al., 2011), and neuroticism (Purves et al., 2020), indicating shared genetic liability between psychiatric disorders. Genetic risk factors may therefore provide useful information about clinical presentation and outcomes, including comorbidity, but to our knowledge this has not yet been explored.

Our study aimed to replicate and extend the NESDA study and prior anxiety comorbidity research in three key ways, within a cohort of individuals with lifetime experience of anxiety and/or depression. First, to replicate the NESDA findings, we compared single disorders and comorbidities among individuals with lifetime diagnoses of MDD and specific anxiety disorders in terms of sociodemographic factors, traumatic experiences, and clinical characteristics. Specifically, we examined differences between: 1) anxiety-anxiety versus single anxiety, 2) anxiety-MDD versus single anxiety, 3) anxiety-anxiety versus anxiety-MDD, and 4) anxiety-MDD versus MDD only. Second, building upon the NESDA findings, we investigated whether associations with anxiety-MDD comorbidity differed by self-reported temporal sequence (i.e., anxiety-first or MDD-first). Third, for a subset of participants with available data, we examined the association of comorbidity groups and temporal sequence with familial risk using two different indices: self-reported family history of mental health diagnoses and genetic factors (i.e., polygenic risk scores). Although analyses of self-reported family history were an additional replication of the NESDA study, this was the first time that genetic factors had been explored in this context.

## 2 Methods

### 2.1 Sample

Data was drawn from the National Institute for Health and Care Research (NIHR) BioResource cohort (*N* = 63,411). This included data from the Genetic Links to Anxiety and Depression (GLAD) Study and the COVID-19 Psychiatry and Neurological Genetics (COPING) study. We utilised data from Data Freeze 1.5, which included all participants who had signed up before 17th August 2021.

The GLAD Study was open to individuals aged 16 or older, living in the UK, with lifetime anxiety and/or depressive disorders. Participants signed up online via the GLAD website (https://gladstudy.org.uk/) where they consented and completed a sign-up questionnaire. They were then sent a saliva DNA kit in the post to their chosen address. See Davies et al. (2019) for a full description of the GLAD recruitment, eligibility, and data collection procedures. Recruitment began in September 2018 and is ongoing.

Recruitment for the COPING study involved email invitations sent to members of the GLAD Study and the general NIHR BioResource (NBR), which includes members of an Inflammatory Bowel Disease cohort and general population cohorts. Participants signed up to the COPING study by following a link and completing an online consent form and questionnaire. All participants of GLAD and NBR were eligible for COPING. Recruitment began in April 2020 and was ongoing until February 2022.

### 2.2 Eligibility and exclusions

For the purposes of this study, we only included GLAD or COPING NBR participants (*N* = 45,639) who fulfilled the DSM-5 diagnostic criteria (American Psychiatric Association, 2013) for a lifetime anxiety disorder or MDD based on symptom responses to the CIDI-SF (Kessler et al., 1998). Participants were excluded for missing data on age, sex, and symptom-based diagnoses which impacted comorbidity group categorisation (see Supplementary Methods 1 for full details). The final sample included 38,775 participants (GLAD *N* = 35,210; COPING NBR *N* = 3565).

#### 2.2.1 Familial risk analyses

The family history and genetic analyses consisted of smaller subsamples of participants who also had family history (*N* = 15,525) or genetic data (*N* = 16,191) available, which were therefore underpowered. Further information about the methods and discussion of the results of these analyses have therefore been moved to Supplementary Methods 2 and Supplementary Results 4a-b, respectively.

### 2.3 Measures

*Lifetime, “symptom-based” disorders* for the comorbidity groups were obtained for MDD, generalised anxiety disorder (GAD), specific phobia, social anxiety disorder, panic disorder, and agoraphobia using the self-report, online version of the Composite International Diagnostic Interview - short form (CIDI-SF; Kessler et al., 1998). Participants' symptom reports were then compared to the Diagnostic and Statistical Manual of Mental Disorders, 5th edition (DSM-5; American Psychiatric Association, 2013) criteria via algorithms which determined their likely diagnostic status (Davies et al., 2022). We use the term symptom-based disorders to emphasise that these variables were derived from participants reporting their symptoms, and to distinguish them from participant self-reporting a diagnosis they received from a health professional (termed “self-reported”). The scripts for these algorithms are available on Github (https://github.com/mollyrdavies/GLAD-Diagnostic-algorithms). “Anxiety” in the current paper refers to those with at least one symptom-based anxiety disorder. These symptom-based disorders were used to define the lifetime comorbidity groups: single anxiety, anxiety-anxiety, anxiety-MDD, and MDD only (see STable 1a for details). The frequency of anxiety disorders which make up each of the anxiety groups (i.e., single anxiety, anxiety-anxiety, and anxiety-MDD) is displayed in STable 1b. Comorbidity groups were defined as comorbidity across the lifespan, and therefore may not be co-occurring.

*Sociodemographic factors* included sex, highest educational attainment (university, A-levels, GCSEs, or no qualifications), age (years), and ethnicity (white or minoritised ethnic group). Individuals with a national vocational qualification (NVQ) or equivalent were included in the GCSEs category to be conservative as we did not collect level information, although we note that NVQ Levels 3 and above are equivalent to A-levels or higher. Ethnicity was also assessed in greater detail, but the categories were combined due to small numbers in each of the minoritised ethnic groups.

*Traumatic experiences* included childhood, adult, and catastrophic trauma.

**Childhood trauma** and **adult trauma** were assessed using the Childhood Trauma Screener (CTS) and Adult Trauma Screener (ATS), respectively (Glaesmer et al., 2013). **Catastrophic trauma**, also referred to as post-traumatic stress disorder (PTSD)-relevant trauma, which assessed experiences of stressful life events after age 16 (Coleman et al., 2020; Davis et al., 2020). Additional details of the measures and scoring are included in Supplementary Methods 3a.

*Clinical characteristics* included self-reported anxiety or depressive disorder diagnosis, other self-reported mental health diagnosis, age of onset, recurrence, current depressive symptoms, and current anxiety symptoms. **Self-reported diagnosis** captured whether participants had ever received a diagnosis from a health professional for an anxiety or depressive disorder, or other mental health diagnosis (STable 2). **Age of onset** was participants' age at the first episode of each disorder, and the earliest age of onset was used in cases of comorbidity. For **recurrence**, participants estimated the number of times they experienced an episode of each disorder on a scale of 1–13+. In cases of comorbidity, the highest reported number of episodes was used. **Current symptoms** were assessed using the 9-item Patient Health Questionnaire (PHQ-9) (Kroenke et al., 2001) and 7-item General Anxiety Disorder assessment (GAD-7) (Spitzer et al., 2006). Further details and scoring are included in Supplementary Methods 3b.

We had planned to include a measure of chronicity. However, the only method available was to calculate the difference between reported ages at first and last episode. This was unable to take into account recurrence, number of episodes, or duration of each episode. Furthermore, high multicollinearity was found between chronicity and both age and age of onset. Chronicity was therefore dropped from the analyses.

### 2.4 Analysis

Multiple logistic regression analyses were conducted to compare differences in associated factors between the comorbidity groups, in line with the statistical methods conducted in the NESDA study to facilitate replication. A false discovery rate (FDR) was used to correct for multiple testing (Benjamini and Hochberg, 1995). For primary analyses, four binary outcome variables were created to compare: 1) anxiety-anxiety versus single anxiety, 2) anxiety-MDD versus single anxiety, 3) anxiety-anxiety versus anxiety-MDD, and 4) anxiety-MDD versus MDD only. The terms “anxiety-MDD” and “MDD only” were used in this paper to clarify that we only had data available for MDD, and not for the other depressive disorders. For the analysis of temporal sequence, we derived a binary variable to compare differences between individuals with “anxiety-first” and “MDD-first” onset, with “MDD-first” as the reference category since anxiety disorders typically have a younger age of onset than depressive disorders (Kessler et al., 2005; Solmi et al., 2022).

Initially, we ran univariate regression analyses (unadjusted models) with each variable regressed independently on each outcome. Then, following the methods of the NESDA paper, three separate regression analyses were run with variables within each of the following categories, referred to as the partially adjusted models: sociodemographic factors, traumatic experiences, and clinical characteristics. Then, all factors were combined into a final model for each comparison, which we refer to as the fully adjusted model. Each logistic regression was performed utilising a complete case analysis for independent variables.

Analyses were pre-registered on the Open Science Framework, accessible at https://osf.io/x3unj/.

#### 2.4.1 Multicollinearity

Multicollinearity refers to instances when an independent variable correlates highly with other independent variables in multiple regression, to the extent where its shared variance can account for much of the relationship with the outcome. This may mask the real relationship between other variables and outcomes, rendering them statistically insignificant, or switching the direction of effect (e.g., of the odds ratio). We examined Pearson correlations between the independent variables (SFigure 2) and used a correlation of 0.80 as a threshold for multicollinearity (Senaviratna and Cooray, 2019). The variance inflation factor (VIF) for each variable was also examined (STable 3a-c).

### 2.5 Code availability

All data cleaning and analyses were conducted using R version 3.5.3 (R Core Team, 2018), the tidyverse (Wickham et al., 2019), corrplot (Wei, 2021), car (Fox and Weisberg, 2018), and stats (R Core Team, 2018) packages. The full code for the analyses included in this paper are available at https://github.com/mollyrdavies/ANXCMBD-GLAD-COPING.

### 2.6 Data availability

GLAD and COPING study data are available via a data request application to the NIHR BioResource (https://bioresource.nihr.ac.uk/using-our-bioresource/academic-and-clinical-researchers/apply-for-bioresource-data/). The data are not publicly available due to restrictions outlined in the study protocol and specified to participants during the consent process. A specific data freeze is available including the variables for the analyses described in this paper; email gladstudy@kcl.ac.uk for details.

## 3 Results

### 3.1 Sample characteristics

**Fig. 1:**
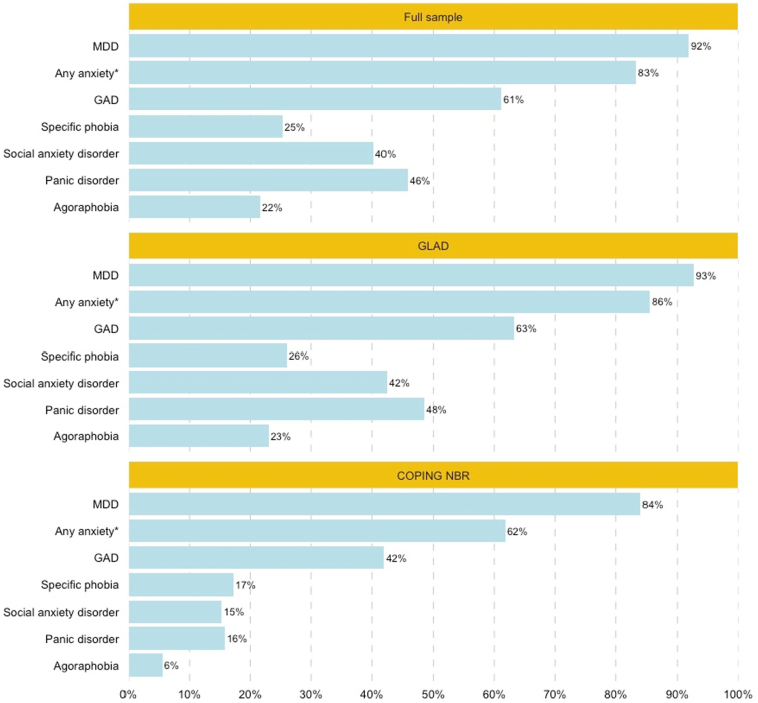
**Frequencies of lifetime major depressive disorder, any anxiety, and the anxiety disorders for the full sample and by cohort.** The bars represent the proportion (%) of the full sample (*N* = 38,775), GLAD (*N* = 35,210), or COPING NBR (*N* = 3565). *“Any anxiety” includes participants with at least one anxiety disorder (GAD, specific phobia, social anxiety disorder, panic disorder, and/or agoraphobia) on the symptom-based measure. Abbreviations: GLAD = Genetics Links to Anxiety and Depression; COPING NBR = COVID-19 Psychiatry and Neurological Genetics NIHR BioResource; MDD = major depressive disorder; GAD = generalised anxiety disorder

Eligible participants (*N* = 38,775) had an average age of 39 years and were predominantly female (79 %). The majority self-identified as white (95 %), and over half had a university degree (56 %). The sample presented as severe with chronic, recurrent symptoms and high instances of comorbidity, although differences were observed between the cohorts. The GLAD cohort had a more severe clinical presentation than COPING NBR. Descriptives of all variables for the full sample and by cohort are displayed in STable 4. Additionally, the majority of participants (75 %) presented with anxiety-MDD comorbidity, compared to 4 % with anxiety-anxiety comorbidity, 5 % with single anxiety, and 17 % with MDD only (STable 1). The frequencies of each of the symptom-based diagnoses or “any anxiety” in the full sample and by cohort are displayed in Fig. 1. MDD was the most common symptom-based diagnosis in the sample (92 %), followed by GAD (61 %).

### 3.2 Anxiety disorder comorbidity comparisons

**Fig. 2:**
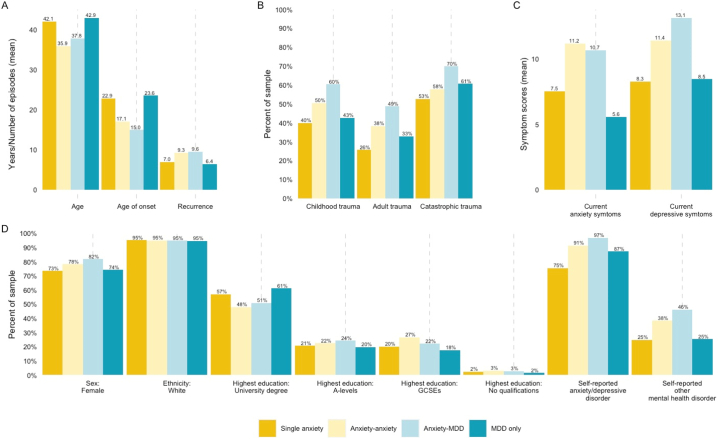
Descriptives of sociodemographic, trauma, and clinical factors by comorbidity group.

Descriptives for each of the risk indicators and clinical characteristics included in this study were examined by comorbidity group and the results are displayed in Fig. 2. Details of the sample size for each model are included in STable 5. Results from the unadjusted (univariate) and the partially adjusted and fully adjusted logistic regression models for all comparisons are displayed in STable 6 in Table 1, respectively. Additional details relating to the results are included in Supplementary Materials, including the discussion of the unadjusted and partially adjusted model findings (Supplementary Results 1a-d). Results from the fully adjusted models are also displayed in a forest plot (SFigure 3).

**Table 1:** **Results from partially adjusted and fully adjusted logistic regression models for the comorbidity group comparisons.** The table displays the odds ratios and confidence intervals for the logistic regression models for each comparison indicated in the column headers. The column headers also indicate the direction of the comparison, with the reference group in parentheses. For example, in the first column “Anx-anx (vs Anx)”, anxiety-anxiety comorbidity is being compared to single anxiety, such that OR > 1 indicates a higher likelihood of having anxiety-anxiety. The partially adjusted models refer to the regressions run with each group of independent variables separately (e.g., sociodemographic variables only). The full models include all three sets of variables. The significance values (q) were adjusted using the false discovery rate multiple testing correction. Note that confidence intervals, while reported, are not fully informative due to the adjustment for multiple testing.

Variable	Partially adjusted models				Fully adjusted models			
	Anx-anx (vs Anx) OR (95 % CI)	Anx-MDD (vs Anx) OR (95 % CI)	Anx-anx (vs Anx-MDD) OR (95 % CI)	Anx-MDD (vs MDD only) OR (95 % CI)	Anx-anx (vs Anx) OR (95 % CI)	Anx-MDD (vs Anx) OR (95 % CI)	Anx-anx (vs Anx-MDD) OR (95 % CI)	Anx-MDD (vs MDD only) OR (95 % CI)
*Sociodemographic*								
Age	0.77⁎⁎⁎ (0.73, 0.81)	0.84⁎⁎⁎ (0.81, 0.87)	0.89⁎⁎⁎ (0.85, 0.92)	0.80⁎⁎⁎ (0.78, 0.82)	0.90⁎ (0.83, 0.97)	1.07 (1.00, 1.14)	0.82⁎⁎⁎ (0.77, 0.86)	1.06⁎⁎⁎ (1.03, 1.09)
Female	1.14 (0.95, 1.35)	1.45⁎⁎⁎ (1.28, 1.64)	0.74⁎⁎⁎ (0.66, 0.84)	1.37⁎⁎⁎ (1.28, 1.47)	1.18 (0.91, 1.54)	1.17 (0.96, 1.42)	0.92 (0.79, 1.08)	1.08 (0.99, 1.18)
University degree	0.51⁎⁎ (0.32, 0.81)	0.71 (0.49, 0.99)	0.84 (0.63, 1.15)	0.39⁎⁎⁎ (0.31, 0.49)	0.79 (0.37, 1.60)	0.98 (0.53, 1.70)	0.92 (0.65, 1.34)	0.81 (0.59, 1.08)
A-levels or equivalent	0.61 (0.37, 0.98)	0.87 (0.59, 1.23)	0.77 (0.57, 1.07)	0.53⁎⁎⁎ (0.42, 0.66)	0.64 (0.30, 1.31)	1.05 (0.57, 1.82)	0.62⁎ (0.43, 0.91)	0.77 (0.56, 1.03)
GCSEs or equivalent	0.90 (0.56, 1.46)	0.95 (0.65, 1.34)	1.08 (0.80, 1.50)	0.64⁎⁎⁎ (0.50, 0.80)	0.69 (0.33, 1.38)	1.08 (0.59, 1.84)	0.60⁎ (0.42, 0.87)	0.66⁎ (0.49, 0.88)
White	1.09 (0.78, 1.53)	1.03 (0.79, 1.30)	1.07 (0.86, 1.37)	1.17⁎⁎ (1.03, 1.33)	1.28 (0.75, 2.15)	0.88 (0.57, 1.31)	1.07 (0.81, 1.44)	1.26⁎⁎ (1.08, 1.47)

*Trauma*								
Childhood trauma	1.39⁎⁎⁎ (1.19, 1.62)	1.78⁎⁎⁎ (1.59, 2.00)	0.78⁎⁎⁎ (0.70, 0.87)	1.79⁎⁎⁎ (1.69, 1.90)	1.10 (0.87, 1.38)	1.18 (0.99, 1.41)	0.91 (0.80, 1.04)	1.11⁎ (1.03, 1.20)
Adult trauma	1.63⁎⁎⁎ (1.38, 1.92)	2.16⁎⁎⁎ (1.91, 2.45)	0.75⁎⁎⁎ (0.67, 0.84)	1.67⁎⁎⁎ (1.57, 1.77)	1.27 (0.99, 1.63)	1.49⁎⁎⁎ (1.23, 1.81)	0.80⁎⁎ (0.69, 0.91)	1.24⁎⁎⁎ (1.15, 1.34)
Catastrophic trauma	1.01 (0.87, 1.19)	1.45⁎⁎⁎ (1.30, 1.63)	0.69⁎⁎⁎ (0.62, 0.78)	1.11⁎⁎⁎ (1.04, 1.18)	1.01 (0.80, 1.27)	1.35⁎⁎ (1.13, 1.60)	0.77⁎⁎⁎ (0.67, 0.88)	1.03 (0.95, 1.11)

*Clinical*								
Self-reported anxiety/depressive disorder diagnosis	3.10⁎⁎⁎ (2.35, 4.10)	7.98⁎⁎⁎ (6.63, 9.58)	0.39⁎⁎⁎ (0.32, 0.49)	2.20⁎⁎⁎ (1.94, 2.48)	2.96⁎⁎⁎ (2.19, 4.03)	8.21⁎⁎⁎ (6.70, 10.05)	0.42⁎⁎⁎ (0.33, 0.53)	2.08⁎⁎⁎ (1.82, 2.38)
Other self-reported mental health diagnosis	1.20 (0.96, 1.51)	1.35⁎⁎⁎ (1.13, 1.62)	0.86⁎ (0.76, 0.98)	1.40⁎⁎⁎ (1.30, 1.51)	1.05 (0.82, 1.34)	1.14 (0.94, 1.38)	0.89 (0.78, 1.01)	1.34⁎⁎⁎ (1.24, 1.45)
Age of onset	0.72⁎⁎⁎ (0.67, 0.78)	0.62⁎⁎⁎ (0.59, 0.66)	1.26⁎⁎⁎ (1.19, 1.34)	0.63⁎⁎⁎ (0.61, 0.65)	0.77⁎⁎⁎ (0.70, 0.84)	0.60⁎⁎⁎ (0.56, 0.65)	1.40⁎⁎⁎ (1.30, 1.50)	0.61⁎⁎⁎ (0.58, 0.63)
Recurrence	1.06⁎⁎⁎ (1.04, 1.09)	1.03⁎⁎ (1.01, 1.05)	1.02⁎⁎ (1.01, 1.04)	1.08⁎⁎⁎ (1.07, 1.09)	1.07⁎⁎⁎ (1.04, 1.10)	1.03⁎ (1.01, 1.05)	1.04⁎⁎⁎ (1.02, 1.05)	1.07⁎⁎⁎ (1.06, 1.08)
Current depressive symptoms (PHQ9)	1.01 (0.99, 1.04)	1.08⁎⁎⁎ (1.06, 1.10)	0.93⁎⁎⁎ (0.92, 0.94)	0.98⁎⁎⁎ (0.97, 0.99)	1.00 (0.98, 1.03)	1.07⁎⁎⁎ (1.05, 1.09)	0.92⁎⁎⁎ (0.91, 0.94)	0.97⁎⁎⁎ (0.97, 0.98)
Current anxiety symptoms (GAD7)	1.08⁎⁎⁎ (1.06, 1.11)	1.00 (0.98, 1.02)	1.08⁎⁎⁎ (1.07, 1.10)	1.15⁎⁎⁎ (1.14, 1.16)	1.08⁎⁎⁎ (1.05, 1.11)	1.00 (0.98, 1.02)	1.08⁎⁎⁎ (1.06, 1.09)	1.15⁎⁎⁎ (1.14, 1.16)

Abbreviations: Anx = single anxiety; Anx-anx = anxiety-anxiety; Anx-MDD = anxiety-MDD; OR = odds ratio; CI = confidence interval; PHQ9 = 9-item patient health questionnaire; GAD7 = 7-item generalised anxiety disorder assessment.

⁎ q < 0.05.

⁎⁎ q < 0.01.

⁎⁎⁎ q < 0.001.

#### 3.2.1 Anxiety-anxiety vs single anxiety

The first group of models compared participants with anxiety-anxiety comorbidity (*N* = 1609) to those with a single anxiety disorder (*N* = 1524). In the fully adjusted model (Table 1, column 5), anxiety-anxiety displayed a more severe and complex clinical presentation than single anxiety, demonstrating significantly higher rates of self-reported anxiety or depressive disorder diagnoses, younger age of onset, higher recurrence rates, and higher current anxiety symptom scores. For example, those with anxiety-anxiety comorbidity were nearly three times as likely to report that a clinician had previously diagnosed them with an anxiety or depressive disorder than those with a single anxiety disorder (91 % vs 75 %, OR = 3.10; CI: 2.35, 4.10). Differences in university degree attainment and trauma, which were significant in the partially adjusted models (Table 1, column 1), were no longer significant in the fully adjusted analyses.

Although the unadjusted model (OR: 1.08; CI: 1.07, 1.10) and descriptive analyses showed higher current depressive symptom severity for anxiety-anxiety than single anxiety (11.2 vs 7.5; Fig. 2), these differences were not significant in either the partially adjusted or adjusted models. To assess whether the correlation between current depressive and anxiety symptoms (*r* = 0.71) might have impacted the significant differences between these groups, a post-hoc analysis was conducted without current anxiety symptoms. The fully adjusted model without current anxiety symptoms demonstrated that anxiety-anxiety had significantly higher depressive symptoms than single anxiety (OR = 1.04; CI: 1.02, 1.07). Results from this model are displayed in STable 7.

#### 3.2.2 Anxiety-MDD vs single anxiety

Participants with anxiety-MDD comorbidity (*N* = 29,161) were then compared to those with a single anxiety disorder (*N* = 1524). After adjusting for all factors (Table 1, column 6), the largest effect was observed for self-reported anxiety or depressive disorder diagnosis. Specifically, individuals with anxiety-MDD were approximately eight times more likely to self-report having been given an anxiety or depressive diagnosis by a clinician than those with single anxiety (OR = 7.98; CI: 6.63, 9.58). This difference is also observable in Fig. 2, which demonstrates that 97 % of individuals with anxiety-MDD report prior experiences of adult trauma compared to 75 % of individuals with single anxiety. Anxiety-MDD also displayed significantly higher rates of reported adult and catastrophic trauma than single anxiety. In terms of clinical characteristics, anxiety-MDD was associated with significantly younger age of onset, higher recurrence, and higher levels of current depressive symptoms. Although sex, childhood trauma, and other self-reported mental health diagnosis were significant in the unadjusted and partially adjusted analyses (STable 6 and Table 1, column 2), they were no longer significant after adjusting for the other factors.

#### 3.2.3 Anxiety-anxiety vs anxiety-MDD

Next, the two comorbidity groups were compared to assess differences between anxiety-anxiety (*N* = 1609) and anxiety-MDD comorbidity (*N* = 29,161). Compared to anxiety-MDD, the fully adjusted analyses found that individuals with anxiety-anxiety were younger, less likely to have A-levels or GCSEs or equivalent than no qualifications, and had lower rates of reported adult and catastrophic trauma. Compared to anxiety-MDD, anxiety-anxiety was also significantly associated with a higher likelihood of self-reporting an anxiety or depressive disorder diagnosis, higher current anxiety symptoms, and lower current depressive symptoms. For instance, individuals with anxiety-anxiety had 8 % higher current anxiety symptom scores than those with anxiety-MDD (11.2 vs 10.7; OR = 1.08; CI: 1.06, 1.09).

For recurrence, the model results (OR = 1.04; CI: 1.02, 1.05) contradicted the unadjusted model results (OR: 0.98; CI: 0.97, 1.00) and descriptive analyses (9.6 vs 9.3 episodes; Fig. 2). Exploratory analyses revealed that the odds ratio for recurrence changed direction when entered into a model with age of onset and current depressive symptoms (further details in Supplementary Results 1c). A follow-up analysis was conducted without controlling for these variables and found no significant difference in recurrence between the groups (STable 8).

#### 3.2.4 Anxiety-MDD vs MDD only

In the final comparison, individuals with anxiety-MDD comorbidity (*N* = 29,161) were compared to those with MDD only (*N* = 6481). In the fully adjusted models (Table 1, column 8), individuals with anxiety-MDD were less likely to have GCSEs or equivalent than no qualification, more likely to self-identify as white, and more likely to report experiences of childhood and adult trauma than those with MDD only. The clinical presentation of anxiety-MDD was significantly more severe and complex than MDD-only across all indicators: anxiety-MDD was associated with a higher likelihood of self-reporting a diagnosis of anxiety or depressive disorders or other mental health disorder, a younger age of onset, higher recurrence, and higher current anxiety symptom scores. For current depressive symptoms, the odds ratio from the model contradicted the unadjusted model results (OR: 1.11; CI: 1.11, 1.12) and descriptive analyses (13.1 vs 8.5). Exploratory analyses found that the direction of effect of current depressive symptoms changed when entered into a model with current anxiety symptoms (further details in Supplementary Results 1d). Additionally, the odds ratio for age at registration changed direction in the fully adjusted models and exploratory analyses revealed that the direction of effect changed when this variable was entered into a model with age of onset.

Two follow-up analyses were conducted to examine the relationship between 1) current depressive and anxiety symptoms and 2) age at registration and age of onset on results. In these analyses, first current anxiety symptoms (STable 7) and then age of onset (STable 9) were excluded from the fully adjusted models. In each case, the direction of effect reverted to match the unadjusted model (STable 6) and descriptives (Fig. 2).

The descriptives for each of the sociodemographic factors, traumatic experiences, and clinical characteristics included in the logistic regression models are displayed by comorbidity group. Single anxiety (*N* = 1524) is displayed in dark yellow, anxiety-anxiety (*N* = 1609) in light yellow, anxiety-MDD in light blue (*N* = 29,161), and MDD only in dark blue (*N* = 6481). The units for the graphs vary depending on the variables. Graph A presents the mean age and age of onset (in years) and the mean number of episodes for recurrence. Graph B displays the proportion (%) of participants in the comorbidity groups who experienced each type of trauma. Graph C shows the mean scores for current anxiety (GAD7) and current depressive (PHQ9) symptoms. Graph D presents the proportion (%) of participants who responded positively to each variable (e.g., for “Sex: Female”, the bars indicate the percent of Female participants).

Abbreviations: MDD = major depressive disorder; GAD7 = 7-item generalised anxiety disorder assessment; PHQ9 = 9-item patient health questionnaire.

#### 3.2.5 Post-hoc sensitivity analysis

The effect size for self-reported anxiety or depressive disorder diagnosis was consistently large in the comparisons between the comorbidity groups. This raised concerns that the lifetime symptom-based disorders (used to assess likely diagnosis for the outcome groups) and the self-reported diagnoses of anxiety and depressive disorders (included as explanatory variables) may be too highly correlated, confounding the models or masking significant associations with other variables. Although prior research from our group demonstrated that the symptom-based and self-reported diagnosis had low to moderate agreement at best (Davies et al., 2022), a sensitivity analysis was conducted to remove self-reported anxiety or depressive disorders from the models. The full results are displayed in STable 10 and described in Supplementary Results 2. In the models excluding self-reported anxiety or depressive disorder diagnosis, anxiety-MDD displayed significantly higher rates of other self-reported mental health diagnoses than single anxiety and anxiety-anxiety, although this difference had not been significant in the previous models. Minor differences between the main results and those of this sensitivity analysis were observed in the significance values of age at registration and sex, but no other differences were found.

### 3.3 Temporal sequence: anxiety-first vs MDD-first

**Fig. 3:**
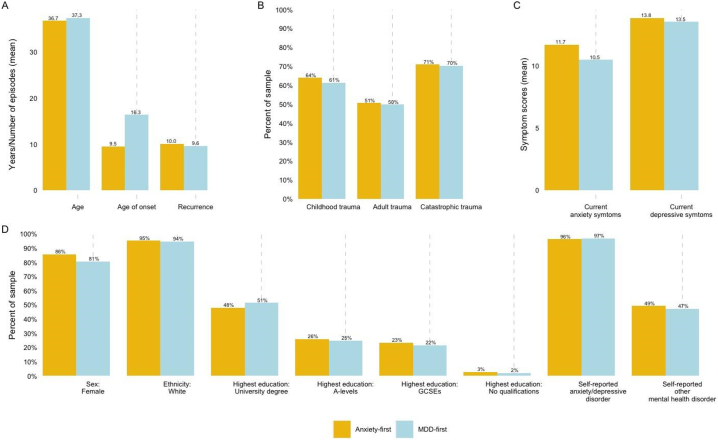
Descriptives by temporal sequence. The descriptives for each of the sociodemographic, trauma, and clinical factors included in the logistic regression model are displayed by anxiety-MDD temporal sequence. Anxiety-first (*N* = 9838) is displayed in yellow, and MDD-first (*N* = 8845) is displayed in blue. The units for the graphs vary depending on the variables. Graph A presents the mean age and age of onset (in years) and the mean number of episodes for recurrence. Graph B displays the proportion (%) of participants in the comorbidity groups who experienced each type of trauma. Graph C shows the mean scores for current anxiety (GAD7) and current depressive (PHQ9) symptoms. Graph D presents the proportion (%) of participants who responded positively to each variable (e.g., for “Sex: Female”, the bars indicate the percent of Female participants). Abbreviations: MDD = major depressive disorder; GAD7 = 7-item generalised anxiety disorder assessment; PHQ9 = 9-item patient health questionnaire

Differences in the temporal sequence of anxiety-MDD were assessed by comparing those with first-onset anxiety disorder (anxiety-first; *N* = 9838) to first-onset MDD (MDD-first; *N* = 8845). Descriptive statistics for each variable were first examined by temporal sequence (Fig. 3). Results from the unadjusted models are displayed in STable 11 and the partially adjusted and fully adjusted logistic regression models are displayed in Table 2. Details of the sample size for each model are included in STable 5. The unadjusted and partially adjusted model results are discussed in Supplementary Results 3a, and SFig. 4 displays the results of the fully adjusted model in a forest plot.

**Table 2:** **Results from partially adjusted and fully adjusted logistic regression models comparing temporal sequence of anxiety-MDD comorbidity.** The table displays the odds ratios and confidence intervals for the logistic regression models to compare first-onset anxiety disorder (anxiety-first) compared to first-onset MDD (MDD-first) in individuals with anxiety-MDD comorbidity. The partially adjusted models refer to the regressions run with each group of independent variables separately (e.g., sociodemographic variables only). The fully adjusted models include all variables, except where specified (i.e., one model did not include age of onset or current anxiety symptoms). The significance values (q) were adjusted using the false discovery rate multiple testing correction. Note that confidence intervals, while reported, are not fully informative due to the adjustment for multiple testing.

Variable	Anxiety-first (vs MDD-first)		
	Partially adjusted model OR (95 % CI)	Fully adjusted model OR (95 % CI)	Fully adjusted model without AOO or GAD7 OR (95 % CI)
*Sociodemographic*			
Age	0.98 (0.96, 1.00)	1.27⁎⁎⁎ (1.23, 1.31)	0.99 (0.97, 1.01)
Female	1.44⁎⁎⁎ (1.33, 1.56)	1.17⁎⁎ (1.05, 1.30)	1.46⁎⁎⁎ (1.33, 1.60)
University degree	0.69⁎⁎⁎ (0.57, 0.84)	1.04 (0.80, 1.36)	0.84 (0.67, 1.06)
A-levels or equivalent	0.76⁎ (0.62, 0.93)	0.94 (0.72, 1.22)	0.81 (0.65, 1.02)
GCSEs or equivalent	0.82 (0.67, 0.99)	0.83 (0.64, 1.07)	0.74⁎ (0.59, 0.92)
White	1.15 (1.01, 1.31)	1.14 (0.97, 1.35)	1.14 (0.98, 1.31)

*Trauma*			
Childhood trauma	1.10⁎⁎ (1.04, 1.18)	0.80⁎⁎⁎ (0.73, 0.87)	1.06 (0.99, 1.14)
Adult trauma	1.01 (0.95, 1.08)	0.86⁎⁎⁎ (0.80, 0.94)	0.93 (0.87, 1.00)
Catastrophic trauma	1.00 (0.93, 1.07)	0.90⁎ (0.83, 0.99)	1.02 (0.94, 1.10)

*Clinical*			
Self-reported anxiety/depressive disorder diagnosis	0.75⁎⁎ (0.62, 0.91)	0.72⁎⁎ (0.58, 0.89)	0.77⁎ (0.64, 0.93)
Other self-reported mental health diagnosis	0.79⁎⁎⁎ (0.74, 0.85)	0.83⁎⁎⁎ (0.77, 0.90)	1.03 (0.96, 1.11)
Age of onset	0.15⁎⁎⁎ (0.14, 0.17)	0.12⁎⁎⁎ (0.11, 0.13)	–
Recurrence	0.98⁎⁎⁎ (0.97, 0.99)	0.96⁎⁎⁎ (0.95, 0.97)	1.03⁎⁎⁎ (1.02, 1.03)
Current depressive symptoms (PHQ9)	0.96⁎⁎⁎ (0.95, 0.97)	0.96⁎⁎⁎ (0.96, 0.97)	1.00 (1.00, 1.01)
Current anxiety symptoms (GAD7)	1.05⁎⁎⁎ (1.05, 1.06)	1.06⁎⁎⁎ (1.05, 1.07)	–

Abbreviations: OR = odds ratio; CI = confidence interval; AOO = age of onset; PHQ9 = 9-item patient health questionnaire; GAD7 = 7-item generalised anxiety disorder assessment.

⁎ q < 0.05.

⁎⁎ q < 0.01.

⁎⁎⁎ q < 0.001.

After adjusting for all factors, participants with anxiety-first were significantly more likely to be female and less likely to hold a university degree, or A-levels or equivalent, than no qualifications compared to those with MDD-first. Clinically, individuals with anxiety-first presented with a younger age of onset, a lower likelihood of self-reporting having received an anxiety or depressive disorder diagnosis by a clinician, and more severe current anxiety symptoms.

Several of the odds ratios in the fully adjusted model contradicted results from the unadjusted and descriptive analyses. Exploratory analyses revealed that the direction of effect for each of these variables changed when entered into a model with age of onset and current anxiety symptoms (full details in Supplementary Results 3b). A sensitivity analysis was therefore run to exclude these variables (fully adjusted model results displayed in Table 2; all model results displayed in STable 12). The directions of effect in the fully adjusted model *without* age of onset and current anxiety symptoms found that anxiety-first had significantly higher recurrence than MDD-first and no significant differences in age at registration, self-reported trauma, other self-reported mental health diagnosis, and current depressive symptoms.

### 3.4 Familial risk

Results from the familial risk analyses are discussed in Supplementary Results 4. Significant differences were found in self-reported family history and genetic risk in the unadjusted and partially adjusted analyses, but none of these differences remained significant in the fully adjusted models. However, after adjusting for all factors, anxiety-anxiety became significantly associated with higher polygenic scores for neuroticism than single anxiety.

## 4 Discussion

Our study represents the largest and most comprehensive investigation of factors associated with anxiety comorbidity to date. We aimed to replicate and extend results from the first in-depth study on anxiety disorder comorbidity by the NESDA group (Hofmeijer-Sevink et al., 2012) and examine differential associations between risk factors and clinical characteristics in 1) anxiety-anxiety vs single anxiety, 2) anxiety-MDD vs single anxiety, 3) anxiety-anxiety vs anxiety-MDD, and 4) anxiety-MDD vs MDD only. Additionally, we examined whether these associations differed for individuals with anxiety-first or MDD-first anxiety-MDD comorbidity. On a subsample of participants, we further explored differences in familial risk measured by self-report of family history or genetic factors (polygenic risk scores).

### 4.1 Differences between the anxiety comorbidity groups

Our findings broadly replicated results from the NESDA paper, demonstrating that the comorbidity groups had a more severe presentation than the single disorders. In comparison to individuals with a single anxiety disorder, those with anxiety-anxiety or anxiety-MDD comorbidity showed higher rates of self-reported anxiety or depressive disorder diagnoses, younger age of onset, and higher recurrence. After removing current anxiety symptoms from our model, we also replicated NESDA findings that anxiety-anxiety comorbidity displayed significantly higher depressive symptoms than single anxiety (Hofmeijer-Sevink et al., 2012). Our unadjusted and descriptive analyses corroborated these results. As such, initially we believed that the interaction between current anxiety and depressive symptoms was evidence of multicollinearity issues biasing model results. However, rather than indicating an issue with the model, it is also possible that the significant difference in current depressive symptoms between anxiety-anxiety and single anxiety is driven by current anxiety symptoms; thus, when the model adjusts for current anxiety symptoms, the differences in current depressive symptoms are no longer significant. Anxiety symptoms may therefore be the primary feature of greater clinical severity in the presentation of anxiety-anxiety comorbidity compared to single anxiety.

Greater clinical severity and complexity across clinical indicators was also observed in anxiety-MDD compared to MDD only. Comparable to the results from the comparison between anxiety-anxiety and single anxiety, the direction of effect of current depressive symptoms and age of onset changed direction from the unadjusted to adjusted models. For current depressive symptoms, exploratory analyses revealed that the odds ratio changed direction when controlling for current anxiety symptoms. Again, this may indicate that the primary feature of symptom severity for anxiety-MDD compared to MDD only are more severe anxiety, rather than depressive, symptoms. For recurrence, the direction of effect changed when controlling for age of onset, which likely indicates that the longer disorder duration for anxiety-MDD is driven by its earlier onset than MDD only.

In line with past research, symptom severity differed between the comorbidity groups: compared to single anxiety, anxiety-anxiety showed significantly higher anxiety but not depressive symptoms, whereas anxiety-MDD displayed significantly higher depressive but not anxiety symptoms. Likewise, anxiety-anxiety comorbidity had significantly higher anxiety symptoms but lower depressive symptoms than anxiety-MDD comorbidity. To our knowledge, this study is the first to demonstrate that anxiety-anxiety comorbidity was associated with higher anxiety symptom severity than anxiety-MDD, as this difference was not significant in the NESDA analyses.

Individuals who reported experiences of trauma were more likely to have anxiety-MDD comorbidity than anxiety-anxiety comorbidity and the single disorders. These results replicated past findings that trauma is more highly associated with MDD than anxiety (Hovens et al., 2010, Hovens et al., 2012; Wiersma et al., 2009) and also with psychiatric comorbidity (Chen et al., 2019; Spinhoven et al., 2010). In the comparison between anxiety-anxiety comorbidity and single anxiety, partially adjusted analyses (which only included the trauma factors) found that reported childhood and adult trauma were significantly associated with anxiety-anxiety comorbidity. This was not found after adjusting for all factors, but this may have been underpowered to detect these differences as the sample size dropped by almost 50 % from the partially adjusted to the fully adjusted model (STable 5).

Individuals with anxiety-anxiety comorbidity were 60 % less likely to self-report having received an anxiety or depressive disorder diagnosis from a clinician than individuals with anxiety-MDD. When this variable was excluded from the model, individuals with anxiety-anxiety were significantly less likely than those with anxiety-MDD to self-report an “other” mental health disorder diagnosis. It is possible that mental health diagnoses overall may be less common for individuals with anxiety-anxiety than anxiety-MDD comorbidity. One possibility is that individuals with anxiety-anxiety comorbidity may have higher functioning, fewer comorbidities with other psychiatric disorders, and/or lower level of risk behaviours (e.g., suicidality). They may therefore not seek or receive help for these symptoms, despite reporting similarly severe symptoms and duration of illness. This would align with NESDA results finding that anxiety-depression was associated with more disability and treatment seeking than anxiety-anxiety. It is well documented that a longer duration of untreated illness is a risk factor for non-response to treatment and increases risk for developing other psychiatric disorders in the future (Altamura et al., 2005, Altamura et al., 2008, Altamura et al., 2013; Bukh et al., 2013; Kraus et al., 2019). Improving early recognition of anxiety disorders, even for individuals with relatively low functional impairment, may thus improve later outcomes for patients and reduce treatment costs.

The NESDA paper also found a higher rate of chronicity for anxiety-anxiety comorbidity compared to anxiety-depression (Hofmeijer-Sevink et al., 2012), which we were unable to assess directly. Chronicity in NESDA was defined as symptoms present for at least 50 % of months in the preceding 4 years, whereas the present analyses only included a measure of recurrence, a clinical feature related to chronicity. However, no significant differences in recurrence were found between the types of comorbidity after adjusting for all factors. Additionally, contrary to NESDA findings, results from the fully adjusted models found no significant difference in age of onset between anxiety-anxiety and anxiety-MDD comorbidity. Ultimately, further research is required to better tease apart the associations between comorbidity and increased risk for symptom severity and duration.

### 4.2 Associations with temporal sequence

We investigated differences between individuals with anxiety-MDD who had a first-onset anxiety disorder (anxiety-first) and those who had first-onset MDD (MDD-first). The strongest observed effect was the difference in age of onset. Anxiety-first displayed a significantly younger age of onset, indicating that a young age of onset is the primary feature of anxiety-first compared to MDD-first. This is consistent with past findings that anxiety disorders have an earlier age of onset than MDD and that first-onset anxiety disorder is more common in cases of comorbidity (Brown et al., 2001; de Graaf et al., 2003; Kessler et al., 2012, Kessler et al., 2015; Lamers et al., 2011). Early interventions to treat anxiety disorders at onset may help to prevent subsequent depressive or other psychiatric symptoms, therefore improving recognition and diagnosis of anxiety disorders should constitute a public health priority.

### 4.3 Familial risk

The family history and genetic analyses had small sample sizes and were thus underpowered. Results from the unadjusted and partially analyses suggested there may be potential differences in familial risk factors, and results from the fully adjusted analysis found that anxiety-anxiety comorbidity was associated with significantly higher polygenic risk for neuroticism than single anxiety. Further research is therefore required to determine whether family history and/or genetic information could be used to assess risk for developing a future comorbid mental health condition at the first onset of an anxiety or depressive disorder.

### 4.4 Limitations

This study has several limitations. First, the sample was majority white, female, and highly educated and therefore not sociodemographically representative of the UK population. The GLAD cohort also displayed a relatively severe and complex clinical presentation, with high rates of comorbidity, reported trauma, and current symptom scores, likely because the GLAD Study specifically recruits individuals with a lifetime history of an anxiety or depressive disorder. It is encouraging that these findings broadly replicated those from the NESDA study, but some findings may not generalise to a less severe population (e.g., cohorts recruited from the general population). This analysis therefore requires replication in a more representative sample, both in terms of sociodemographic characteristics and severity and complexity of disorder presentation. Furthermore, the use of self-report, retrospective measures enabled recruitment of a large number of individuals, but has the potential for inaccurate or biased recall (Coughlin, 1990; Dunlop et al., 2019). In particular, there may be greater error in reporting age of onset and number of episodes (recurrence) for individuals whose lifetime anxiety or depressive disorder occurred many years prior. The symptom-based measures of anxiety disorders and MDD included in this study require further validation, as some studies have found low agreement between the self-report CIDI-SF and structured interviews (Carlbring et al., 2002). In addition, for the younger people in our sample aged 16–25, many may not have finished education, potentially confounding our analyses of educational attainment. We also did not have adequate measures of chronicity, treatment, disability, or personality traits collected in our sample, and thus were unable to replicate NESDA findings on these factors. Although we examined familial risk with a subsample of participants with available data, these analyses were likely underpowered to detect significant effects. Moreover, the predictive utility of polygenic risk scores is limited by the power of the GWAS from which they were derived. Many of the polygenic scores in this analysis, particularly for the psychiatric disorders, only explain a small proportion of the heritability of the corresponding phenotype. The sample sizes and corresponding power of GWAS have been steadily increasing over time, therefore these scores may become more effective in the future. Finally, there was a relatively small sample of individuals without MDD, therefore comparisons between anxiety-anxiety comorbidity and single anxiety disorder may have been underpowered in the fully adjusted models.

### 4.5 Summary

Overall, anxiety-anxiety comorbidity displayed an equally severe profile to anxiety-MDD in terms of symptom severity and duration of illness. Notably, anxiety-anxiety and anxiety-MDD exhibited similar associations when compared to single anxiety. Despite these similarities, our results also support NESDA findings that anxiety-anxiety and anxiety-MDD comorbidity are clinically distinct, with anxiety-anxiety associated with significantly higher anxiety symptoms but lower depressive symptoms than anxiety-MDD. We did not replicate the NESDA finding that anxiety-anxiety was associated with an earlier age of onset than anxiety-MDD, thus requiring further investigation. For individuals with anxiety-MDD, first-onset anxiety was associated with an earlier age of onset and a more severe clinical presentation. However, anxiety-anxiety comorbidity was associated with lower rates of self-reported diagnoses from health professionals. Prior research has demonstrated that anxiety disorders are typically associated with less recognition and/or treatment-seeking. Increasing early recognition and intervention of anxiety disorders could improve patient outcomes, prevent a later onset of MDD or other psychiatric disorders, and reduce economic and treatment costs. Anxiety disorders and anxiety-anxiety comorbidity are clinically relevant phenotypes which warrant further investigation and attention in research, practice, and public health.

## Funding

This work was supported by the National Institute of Health and Care Research (10.13039/501100000272NIHR) BioResource [RG94028, RG85445], 10.13039/100014461NIHR Biomedical Research Centre [IS-BRC-1215-20018], HSC R&D Division, Public Health Agency [COM/5516/18], MRC Mental Health Data Pathfinder Award (MC_PC_17,217), and the National Centre for Mental Health funding through Health and Care Research Wales. Dr. Goldsmith receives funding from 10.13039/501100000272NIHR, 10.13039/501100000265MRC, 10.13039/100014013UKRI, 10.13039/501100012264NIH, and the 10.13039/100008664Juvenile Diabetes Research Foundation (JDRF). Dr. Krebs is funded by a Clinical Research Training Fellowship from the 10.13039/501100000265Medical Research Council (MR/N001400/1). Brett N Adey is funded through a Pre-doctoral Fellowship from the 10.13039/501100000272NIHR (NIHR301067). Dr. Morneau-Vaillancourt is funded by a Postdoctoral Research Fellowship from the 10.13039/501100000155Social Sciences and Humanities Research Council of Canada (756-2021-0516) and 10.13039/100008240Fonds de recherche du Québec société et culture (2022-B3Z-297753). Dr. Hübel acknowledges funding by 10.13039/501100003554Lundbeckfonden (R276-2018-4581). Jessica Mundy acknowledges funding from the Lord Leverhulme Charitable Grant.

## Conflict of interest

Prof Breen has received honoraria, research or conference grants and consulting fees from Illumina, Otsuka, and COMPASS Pathfinder Ltd. Prof Hotopf is principal investigator of the RADAR-CNS consortium, an IMI public private partnership, and as such receives research funding from 10.13039/100005205Janssen, 10.13039/100011944UCB, 10.13039/100005614Biogen, 10.13039/501100013327Lundbeck and 10.13039/100009947MSD. Prof McIntosh has received research support from 10.13039/100004312Eli Lilly, 10.13039/100005205Janssen, and the Sackler Foundation, and has also received speaker fees from Illumina and Janssen. Prof Walters has received grant funding from Takeda for work unrelated to the GLAD Study. The remaining authors have nothing to disclose.

## Appendix A Supplementary data

Supplementary material Image 1
