# Supplementary material for: Factors associated with anxiety disorder comorbidity
Source: J Affect Disord. 2023 Feb 15;323:280–91. doi: 10.1016/j.jad.2022.11.051 (PMC10202820; doi:10.1016/j.jad.2022.11.051)
Supplement: Supplementary file 1 — Supplementary material [file mmc1.docx]

**Factors associated with anxiety disorder comorbidity**

Supplementary materials

**Authors:** Molly R. Davies^a,b orcid.org/0000-0003-3483-9907^, Kiran Glen^a,b orcid.org/0000-0002-2831-3266^, Jessica Mundy^a,b 0000-0001-5513-8902^, Abigail R. ter Kuile^a,b orcid.org/0000-0002-7869-3754^, Brett N. Adey^a,b orcid.org/0000-0003-4356-4079^, Chérie Armour^c orcid.org/0000-0001-7649-3874^, Elham Assary^a orcid.org/^, Jonathan R.I. Coleman^a,b orcid.org/0000-0002-6759-0944^, Kimberley A. Goldsmith^a,b orcid.org/0000-0002-0620-7868^, Colette R. Hirsch^a,b,d orcid.org/0000-0003-3579-2418^, Matthew Hotopf^a,b,d orcid.org/0000-0002-3980-4466^, Christopher Hübel^a,b,e,f orcid.org/0000-0002-1267-8287^, Ian R. Jones^g orcid.org/0000-0001-5821-5889^, Gursharan Kalsi^a,b orcid.org/0000-0002-5156-7176^, Georgina Krebs^a,h orcid.org/0000-0002-5353-5645^, Andrew M. McIntosh^i orcid.org/0000-0002-0198-4588^, Geneviève Morneau-Vaillancourt^a orcid.org/0000-0002-5322-6252^, Alicia J. Peel^a orcid.org/0000-0002-6144-5412^, Kirstin L. Purves^a,b orcid.org/0000-0002-8110-5554^, Sang Hyuck Lee^a,b orcid.org/0000-0002-5610-033X^, Megan Skelton^a,b orcid.org/0000-0002-7619-349X^, Daniel J. Smith^i orcid.org/0000-0002-2267-1951^, David Veale^a,b,d orcid.org/0000-0002-1717-4766^, James T. R. Walters^g orcid.org/0000-0002-6980-4053^, Katherine S. Young^a,b orcid.org/0000-0002-1378-6415^, Johan Zvrskovec^a,b orcid.org/0000-0002-8862-0874^, Gerome Breen^a,b orcid.org/0000-0003-2053-1792^, Thalia C. Eley * ^a,b orcid.org/0000-0001-6458-0700^

^a^Institute of Psychiatry, Psychology and Neuroscience, King's College London, Denmark Hill, Camberwell, London, UK

^b^National Institute for Health and Care Research (NIHR) Biomedical Research Centre, South London and Maudsley Hospital, London, UK

^c^Stress, Trauma & Related Conditions (STARC) research lab, School of Psychology, Queen's University Belfast (QUB), Belfast, Northern Ireland, UK

^d^South London and Maudsley NHS Foundation Trust, Denmark Hill, Camberwell, London, UK

^e^Department of Medical Epidemiology and Biostatistics, Karolinska Institutet, Stockholm, Sweden

^f^National Centre for Register-based Research, Aarhus Business and Social Sciences, Aarhus University, Aarhus, Denmark

^g^National Centre for Mental Health, Division of Psychiatry and Clinical Neuroscience, Cardiff University, Cardiff, UK

^h^Research Department of Clinical, Educational and Health Psychology, University College London, 1-19 Torrington Place, London, UK

^i^Division of Psychiatry, Centre for Clinical Brain Sciences, University of Edinburgh, Edinburgh, UK

*Corresponding author: Prof. Thalia C Eley (thalia.eley@kcl.ac.uk, +442078480863), Social, Genetic and Developmental Psychiatry Centre, Institute of Psychiatry, Psychology and Neuroscience, King’s College London, London, SE5 8AF, UK

Table of Contents

[Supplementary Methods 5](#_Toc115777536)

[Supplementary Methods 1. Eligibility and exclusions. 5](#_Toc115777537)

[Supplementary Methods 2. Familial risk analyses 6](#_Toc115777538)

[Supplementary Methods 2a Sample 6](#_Toc115777539)

[Supplementary Methods 2b Measures 6](#_Toc115777540)

[Supplementary Methods 2b.1 Self-reported family history 6](#_Toc115777541)

[Supplementary Methods 2b.2 Genetic factors 6](#_Toc115777542)

[Supplementary Methods 2c. Analyses 7](#_Toc115777543)

[Supplementary Methods 3. Measures and scoring. 8](#_Toc115777544)

[Supplementary Methods 3a Traumatic experiences. 8](#_Toc115777545)

[Supplementary Methods 3b. Clinical characteristics 8](#_Toc115777546)

[Supplementary Results 9](#_Toc115777547)

[Supplementary Results 1. Results from the partially adjusted analyses for the comorbidity groups 9](#_Toc115777548)

[Supplementary Results 1a. Anxiety-anxiety vs single anxiety 9](#_Toc115777549)

[Supplementary Results 1b. Anxiety-MDD vs single anxiety 9](#_Toc115777550)

[Supplementary Results 1c. Anxiety-anxiety vs anxiety-MDD 10](#_Toc115777551)

[Supplementary Results 1d. Anxiety-MDD vs MDD only 10](#_Toc115777552)

[Supplementary Results 2. Post-hoc sensitivity analyses for the comorbidity group comparisons 11](#_Toc115777553)

[Supplementary Results 3. Temporal sequence: anxiety-first vs MDD-first 12](#_Toc115777554)

[Supplementary Results 3a. Temporal sequence partially adjusted analysis results 12](#_Toc115777555)

[Supplementary Results 3b. Post-hoc analyses to assess relationship between variables 12](#_Toc115777556)

[Supplementary Results 4. Familial risk 13](#_Toc115777557)

[Supplementary Results 4a. Self-reported family history 13](#_Toc115777558)

[Supplementary Results 4b. Genetic factors 13](#_Toc115777559)

[References 15](#_Toc115777560)

[Supplementary Tables 17](#_Toc115777561)

[STable 1a. Anxiety and MDD comorbidity grouping definitions and frequencies. 17](#_Toc115777562)

[STable 1b. Anxiety disorder frequencies by anxiety comorbidity group. 17](#_Toc115777562)

[STable 2. Definitions for family history and self-reported diagnoses. 18](#_Toc115777563)

[STable 3a. Variance inflation factor (VIF) results for comorbidity group and temporal sequence comparisons. 19](#_Toc115777564)

[STable 3b. Variance inflation factor (VIF) results for comorbidity group comparisons, including family history variables. 20](#_Toc115777565)

[STable 3c. Variance inflation factor (VIF) results for comorbidity group comparisons, including genetic factors. 21](#_Toc115777566)

[STable 4. Descriptives for full sample and by cohort. 23](#_Toc115777567)

[STable 5. Sample size for each model. 25](#_Toc115777568)

[STable 6. Results from unadjusted logistic regression models for the comorbidity group comparisons. 26](#_Toc115777569)

[STable 7. Results from the fully adjusted logistic regression model for anxiety-anxiety vs single anxiety and anxiety-MDD vs MDD only, excluding current anxiety symptoms. 28](#_Toc115777570)

[STable 8. Results from the fully adjusted logistic regression model for anxiety-anxiety vs anxiety-MDD, excluding age of onset and current depressive symptoms. 29](#_Toc115777571)

[STable 9. Results from the fully adjusted logistic regression model for anxiety-MDD vs MDD only, excluding age of onset. 30](#_Toc115777572)

[STable 10. Results from partially adjusted and fully adjusted logistic regression models for comorbidity group comparisons, excluding self-reported anxiety or depressive disorder diagnosis. 31](#_Toc115777573)

[STable 11. Results from the unadjusted logistic regression models for temporal sequence comparison. 33](#_Toc115777574)

[STable 12. Results from partially adjusted and fully adjusted logistic regression models for temporal sequence comparison, excluding age of onset and current anxiety symptoms. 34](#_Toc115777575)

[STable 13. Results from partially adjusted and fully adjusted logistic regression models for comorbidity group comparisons, including self-reported family history. 35](#_Toc115777576)

[STable 14. Results from partially adjusted and fully adjusted logistic regression models for temporal sequence comparisons, including self-reported family history. 38](#_Toc115777577)

[STable 15. Results from partially adjusted and fully adjusted logistic regression models for temporal sequence comparisons, including self-reported family history and excluding age of onset and current anxiety symptoms. 39](#_Toc115777578)

[STable 16. Results from partially adjusted and fully adjusted logistic regression models for comorbidity group comparisons, including genetic factors. 40](#_Toc115777579)

[STable 17. Results from partially adjusted and fully adjusted logistic regression models for temporal sequence comparisons, including genetic factors. 45](#_Toc115777580)

[STable 18. Results from partially adjusted and fully adjusted logistic regression models for temporal sequence comparisons, including genetic factors and excluding age of onset and current anxiety symptoms. 47](#_Toc115777581)

[Supplementary Figures 49](#_Toc115777582)

[SFigure 1. Flowchart of genotyping quality control, imputation, and data exclusions 49](#_Toc115777583)

[SFigure 2. Correlation matrix of all independent variables. 50](#_Toc115777584)

[SFigure 3. Forest plot of results from the full logistic regression models for the comorbidity group comparisons. 51](#_Toc115777585)

[SFigure 4. Forest plot of results from the full logistic regression model for the temporal sequence comparison. 53](#_Toc115777586)

[SFigure 5. Descriptives for self-reported family history by comorbidity group. 54](#_Toc115777587)

[SFigure 6. Descriptives for self-reported family history by temporal sequence. 55](#_Toc115777588)

[SFigure 7. Descriptives for genetic factors by comorbidity group. 56](#_Toc115777589)

[SFigure 8. Descriptives for genetic factors by temporal sequence. 57](#_Toc115777590)

# Supplementary Methods

## Supplementary Methods 1. Eligibility and exclusions.

Participants were excluded from analyses for missing data on age (N = 3) or symptom-based diagnoses which had a direct impact on which comorbidity group they would have been categorised in (N = 6,862). For example, if a participant had one anxiety disorder and missing data for MDD, it was not clear whether they should belong to the single anxiety group or the anxiety-MDD group and therefore they were excluded. As such, participants 1) with missing data for an MDD diagnosis or 2) who met criteria for none or one anxiety diagnosis and were missing data for at least one anxiety diagnosis were excluded from analyses. No eligible participants were missing data for sex. Genetic duplicates across GLAD and COPING NBR (N = 31) were identified and one of the duplicate individuals was dropped at random.

##

## Supplementary Methods 2. Familial risk analyses

### Supplementary Methods 2a Sample

The familial risk analyses consisted of smaller subsamples of participants who also had family history (N = 18,388) or genetic data (N = 16,139) available. Additional exclusions were applied to the genetic analyses and are detailed below. The stages of genetic data cleaning and number of participants excluded at each stage are displayed in SFigure 1.

### Supplementary Methods 2b Measures

#### Supplementary Methods 2b.1 Self-reported family history

*Self-reported family history* of mental health disorders was collected in the COPING study baseline questionnaire and as an optional questionnaire in the GLAD Study. Data on these measures was therefore only available for GLAD participants that had enrolled in COPING or had taken part in the optional questionnaire. This data was available for all NBR participants. Participants reported whether any of their immediate family members had been diagnosed with a psychiatric disorder. Responses were converted to binary variables representing the presence or absence of a family history of 1) any anxiety or depressive disorder or 2) other psychiatric disorder (STable 2).

#### Supplementary Methods 2b.2 Genetic factors

Genetic factors*,* or genetic liability*,* included *polygenic risk scores* for disorders and traits that had previously been associated with anxiety disorders.

*Genotyping*

Genotyping of the GLAD and COPING NBR was performed using the ThermoFisher Affymetrix UK Biobank Axiom Array v1 and v2 across numerous genotyping batches. Quality assurance measures were first calculated by ThermoFisher. Samples were retained if they had a dish QC value ≥0.82 (capturing the resolution of true signal from background noise on the genotyping array) and an initial call rate ≥0.97. Variants genotyped with high resolution, classified by ThermoFisher as "PolyHighResolution", "NoMinorHom", or "MonoHighResolution", were recommended for inclusion. Data which passed these initial quality assurance checks were transferred to the NIHR BioResource Centre Maudsley team at King’s College London and underwent further quality control, adapted from previous pipelines [PMID: 26443613].

*Imputation and merging*

Pre-phasing (statistical estimation of haplotypes) was conducted using EAGLE2 [PMID: 27694958]. Data was imputed to the full TopMED panel and then restricted to variants with minor allele frequency (MAF) ≥ 0.001, Hardy-Weinberg equilibrium p<10-10, and imputation R2 ≥ 0.3. Post-imputation VCF files were updated to include sex information and rsIDs, collected from the Single Nucleotide Polymorphism Database, build 153. Post-imputation GLAD and COPING NBR data were merged using bcftools and converted to PLINK2 pfile format, retaining genotype dosage information for bi-allelic SNPs only [PMID: 25722852].

*Variant and individual exclusions*

SNPs with a MAF lower than 0.01 and variant missingness greater than 0.05 were excluded. Samples with mismatched sex, individual missingness greater than 0.05, related individuals with pihat > 0.1875, and heterozygosity outliers were also excluded (SFigure 1)

*Polygenic risk scores*

All polygenic scores were calculated using PRS-CS (Ge et al., 2019) and PLINK 1.9 (Chang et al., 2015). The scores were generated using a reference panel and linkage disequilibrium (LD) scores based on the European UK Biobank cohort and genome-wide association study (GWAS) summary statistics from GWAS for anxiety (Purves et al., 2020), MDD (Wray et al., 2018), neuroticism (Nagel et al., 2018), schizophrenia (Schizophrenia Working Group of the Psychiatric Genomics Consortium, 2014), ADHD (Demontis et al., 2019), ASD (Grove et al., 2019), and educational attainment (Lee et al., 2018).

### Supplementary Methods 2c. Analyses

To assess differences in familial risk, two partially adjusted and two fully adjusted logistic regression analyses were run for each comparison on subsamples of participants with 1) self-reported family history and 2) genetic data. Each logistic regression was performed utilising a complete case analysis for independent variables. Genotyping batch and principal components were added as covariates in models which included the genetic factors.

## Supplementary Methods 3. Measures and scoring.

### Supplementary Methods 3a Traumatic experiences.

**Childhood trauma** and **adult trauma** were assessed using the Childhood Trauma Screener (CTS) and Adult Trauma Screener (ATS), respectively, with 5-point Likert items according to reported frequency of experiences of abuse before age 16 (childhood) or domestic abuse after age 16 (adult). Both measures evaluated physical, emotional, and sexual abuse; childhood trauma also included emotional and physical neglect. The CTS defined cut-off points for each domain which were used to establish presence or absence of the traumatic experience (Glaesmer et al., 2013) and were applied to the corresponding adult items (e.g., the cut-off for childhood sexual abuse was utilised for adult sexual abuse). **Catastrophic trauma**, also referred to as post-traumatic stress disorder (PTSD)-relevant trauma, was measured using six items with binary response options asking whether participants had experienced any of the following stressful life events after age 16: sexual assault, being attacked, mobbed, or robbed, life-threatening accident, life-threatening illness, witnessing a violent death, and exposure to combat or a war zone (Coleman et al., 2020; Davis et al., 2020). Responses to all three trauma measures were converted to binary variables indicating presence or absence of any childhood, adult, or catastrophic trauma.

### Supplementary Methods 3b. Clinical characteristics

**Self-reported diagnosis** captured whether participants had ever received a diagnosis from a health professional for an anxiety or depressive disorder, or other mental health diagnosis. Participants who self-reported having received one or more anxiety and/or depressive disorder diagnoses were classified as having a “**self-reported anxiety or depressive disorder diagnosis**”. Those who reported any other mental health diagnosis were classified as having an “**other self-reported mental health diagnosis**” (STable 2). **Age of onset** was participants’ age at the first episode of each disorder. In cases of comorbidity, the earliest age was used as a measure of age of onset. For **recurrence**, participants estimated the number of times they experienced an episode of each disorder on a scale of 1-13+. In cases of comorbidity, the highest reported number of episodes was used. **Current symptoms** were assessed using the 9-item Patient Health Questionnaire (PHQ-9) (Kroenke et al., 2001) and 7-item General Anxiety Disorder assessment (GAD-7) (Spitzer et al., 2006). Responses were summed into total scores (PHQ-9: 0-27; GAD-7: 0-21) representing the severity of depressive or anxiety symptoms respectively, experienced in the last two weeks.

# Supplementary Results

## Supplementary Results 1. Results from the partially adjusted analyses for the comorbidity groups

### Supplementary Results 1a. Anxiety-anxiety vs single anxiety

The unadjusted models found significant differences for all variables except educational attainment and ethnicity (STable 6, column 1). Results from the partially adjusted models (Table 1, column 2) indicated that participants with anxiety-anxiety were significantly younger, less likely to have a university degree than no qualifications, and more likely to have reported experiences of childhood and adult trauma than those with a single anxiety disorder. Anxiety-anxiety also displayed significantly higher rates of self-reported anxiety or depressive disorder diagnoses, younger age of onset, higher recurrence rates, and higher current anxiety symptom scores, with the largest effect observed for self-reported diagnosis of anxiety or depressive disorder (91% vs 75%; OR = 3.10; CI: 2.35, 4.10). Although the unadjusted models found that anxiety-anxiety was significantly associated with being female, higher rates of catastrophic trauma, higher rates of self-reported “other” mental health disorder diagnosis, and more severe current depressive symptoms compared to single anxiety, these were not significant in the respective partially adjusted models.

### Supplementary Results 1b. Anxiety-MDD vs single anxiety

Similar to the anxiety-anxiety and single anxiety comparison, all factors except educational attainment and ethnicity were significantly different in the unadjusted models (STable 6, column 2). In the partially adjusted models (Table 1, column 2), all factors and characteristics from the unadjusted analyses, except current anxiety symptoms, remained significant after correcting for multiple testing. For example, compared to those with single anxiety, participants with anxiety-MDD were significantly younger, with an odds ratio of 0.84 (CI: 0.81, 0.87). They were also more likely to be female, and more likely to have reported each type of trauma. In the partially adjusted model of clinical characteristics, anxiety-MDD also showed a more severe and complex clinical presentation than single anxiety disorder on most measures, with higher rates of self-reported diagnosis of an anxiety or depressive disorder or other mental health disorder, younger age of onset, more recurrent episodes, and higher current depressive symptom scores. Across the partially adjusted models, the largest difference was observed for self-reported anxiety or depressive disorder diagnosis; individuals with anxiety-MDD were approximately eight times more likely to self-report an anxiety or depressive disorder diagnosis than those with single anxiety (OR = 7.98; CI: 6.63, 9.58). This difference is also observable in Figure 2, which demonstrates that 97% of individuals with anxiety-MDD report prior experiences of adult trauma compared to 75% of individuals with single anxiety.

### Supplementary Results 1c. Anxiety-anxiety vs anxiety-MDD

Unadjusted analyses found significant differences for all factors except educational attainment and ethnicity (STable 6, column 3), which all retained significance in the partially adjusted models. Results from the partially adjusted models (Table 1, column 3) found that individuals with anxiety-anxiety comorbidity were significantly younger, less likely to have GCSEs or equivalent than no educational qualifications, and less likely to self-report experiences of trauma than anxiety-MDD. Clinically, anxiety-anxiety exhibited significantly lower rates of self-reported diagnoses for anxiety or depressive disorders or an “other” mental health disorder, higher age of onset, and less severe current depressive symptoms than anxiety-MDD. By contrast, current anxiety symptoms were more severe for individuals with anxiety-anxiety than those with anxiety-MDD.

Results for recurrence in both the partially adjusted (OR = 1.02; CI: 1.01, 1.04) and fully adjusted (OR = 1.04; CI: 1.02, 1.05) models suggested that individuals with anxiety-anxiety had more recurrent episodes than those with anxiety-MDD, which contradicted the unadjusted model results (OR: 0.98; CI: 0.97, 1.00) and descriptive analyses displayed in Figure 2 (9.6 vs 9.3 episodes). A post-hoc exploratory analysis was conducted to investigate, in which recurrence was entered into a regression model with the other independent variables one at a time. This revealed that the odds ratio for recurrence changed direction when entered into a model with age of onset or current depressive symptoms. A follow-up adjusted logistic regression analysis comparing anxiety-anxiety and anxiety-MDD was conducted without controlling for age of onset and current depressive symptoms, which found no significant difference in recurrence between the groups (STable 8).

### Supplementary Results 1d. Anxiety-MDD vs MDD only

The unadjusted model results found significant differences in all factors except ethnicity (STable 6, column 4). In the partially adjusted analyses (Table 1, column 4) of sociodemographic characteristics, participants with anxiety-MDD were significantly younger, more likely to be female, and less likely to have an educational qualification (i.e., university degree, A-level, GCSEs, or their equivalents). Individuals with anxiety-MDD are also more likely to self-identify as white, a difference which was not significant in the unadjusted models. In the unadjusted models and partially adjusted trauma model, those with anxiety-MDD had a higher likelihood of reporting experiences of trauma than those with MDD only. In terms of clinical characteristics, the unadjusted and partially adjusted model found that anxiety-MDD was associated with a higher likelihood of self-reporting a diagnosis of anxiety or depressive disorders or other mental health disorder, a younger age of onset, higher recurrence, and higher current anxiety symptom scores.

For current depressive symptoms, the odds ratio from the model contradicted the unadjusted model results (OR: 1.11; CI: 1.11, 1.12) and descriptive analyses (13.1 vs 8.5). A post-hoc exploratory analysis was conducted to investigate, in which current depressive symptoms were regressed on the outcome with the other independent variables one at a time. Results from these analyses found that the direction of effect for current depressive symptoms changed when entered into a model with current anxiety symptoms. Additionally, the odds ratio for age at registration changed direction in the fully adjusted models compared to the unadjusted and partially adjusted models (STable 6 and Table 1, column 4). Another exploratory analysis was conducted in which age at registration was entered into models with each independent variable one at a time and revealed that the direction of effect of age at registration changed when entered into a model with age of onset. As described in the main manuscript, two follow-up analyses were conducted to examine the relationship between 1) current depressive and anxiety symptoms and 2) age at registration and age of onset. In these analyses, first current anxiety symptoms (STable 7) and then age of onset (STable 9) were excluded from the fully adjusted models. In each case, the direction of effect reverted back to match the unadjusted model (STable 6) and descriptives (Figure 2).

## Supplementary Results 2. Post-hoc sensitivity analyses for the comorbidity group comparisons

As described in the main manuscript, a post-hoc sensitivity analysis was conducted to remove self-reported anxiety or depressive disorders from the models (STable 10). Results from the partially adjusted models without the self-reported anxiety or depressive disorder diagnosis variable were identical for all comparisons except anxiety-anxiety and single anxiety, with minor differences in OR values but no changes in direction of effect or significance than the models with the variable. In the model without self-reported diagnosis of an anxiety or depressive disorder, anxiety-anxiety had higher rates of self-reported “other” mental health diagnoses and higher current depressive symptoms. Fully adjusted model results were also similar, with differences in significance for one or two variables in each model. In the models excluding self-reported anxiety or depressive disorder diagnosis, anxiety-MDD displayed significantly higher rates of other self-reported mental health diagnoses than single anxiety and anxiety-anxiety, although this difference had not been significant in the previous models. In the model without self-reported anxiety or depressive disorder, age at registration was no longer significant in the comparison between anxiety-MDD and single anxiety, and became significantly different between anxiety-anxiety and single anxiety, with anxiety-anxiety being associated with younger age. Finally, individuals with anxiety-MDD were significantly more likely to be female than MDD only. No other differences between the main results and those of this sensitivity analysis were observed.

## Supplementary Results 3. Temporal sequence: anxiety-first vs MDD-first

### Supplementary Results 3a. Temporal sequence partially adjusted analysis results

In the unadjusted and partially adjusted models, significant differences were found for a majority of the factors and characteristics after correcting for multiple testing. Compared to participants with MDD-first, those with anxiety-first were significantly more likely to be female, less likely to hold a university degree or A-levels or equivalent than no qualifications, and more likely to report experiencing childhood trauma. In the unadjusted models, individuals with anxiety-first were also more likely to self-identify as White, but this difference was not significant in the partially adjusted sociodemographic model. Clinically, results from both the unadjusted and partially adjusted models found that individuals with anxiety-first presented with a younger age of onset and more severe current anxiety symptoms than those with MDD-first. In the partially adjusted model, anxiety-first was also associated with a lower likelihood of self-reporting having received an anxiety or depressive disorder diagnosis from a clinician than MDD-first, although this difference was not significant in the unadjusted model. The odds ratios for other self-reported mental health diagnoses (49% vs 47%; OR = 0.79; CI: 0.74, 0.85), recurrence (10.0 vs 9.6; OR = 0.98; CI: 0.97, 0.99) and current depressive symptoms (13.8 vs 13.5; OR = 0.96; CI: 0.95, 0.97) contradicted results from the unadjusted analyses. Follow-up analyses revealed that the direction of effect for current depressive symptoms changed when current anxiety symptoms were included, and all three variables changed direction when age of onset was introduced into the model (discussed further below).

### Supplementary Results 3b. Post-hoc analyses to assess relationship between variables

In the fully adjusted analyses, the odds ratios for other self-reported mental health diagnoses (49% vs 47%; OR = 0.83; CI: 0.77, 0.90), recurrence (10.0 vs 9.6; OR = 0.96; CI: 0.95, 0.97) and current depressive symptoms (13.8 vs 13.5; OR = 0.96; CI: 0.96, 0.97) contradicted results from the unadjusted and descriptive analyses. Moreover, age at registration and adult and catastrophic trauma variables were non-significant in the partially adjusted analyses but became statistically significant in the final model. Follow-up analyses revealed that the directions of effect for current depressive symptoms and adult trauma changed when current anxiety symptoms were included, and all variables changed direction when age of onset was introduced into the model.

Due to the impact of age of onset and current anxiety symptoms on the model results, we ran a sensitivity analysis to exclude these variables (results of the fully adjusted model are displayed in Table 2; results from both partially and fully adjusted models are displayed in STable 12). The directions of effect in the fully adjusted model *without* age of onset and current anxiety symptoms found that anxiety-first had significantly higher recurrence than MDD-first and no significant differences in age at registration, self-reported trauma, other self-reported mental health diagnoses, and current depressive symptoms.

## Supplementary Results 4. Familial risk

### Supplementary Results 4a. Self-reported family history

Results from the unadjusted and the partially and fully adjusted logistic regression models comparing differences in self-reported family history for the comorbidity groups are displayed respectively in STable 6 and STable 13, and for the temporal sequence comparison in STables 11 and 14. Results from the unadjusted and partially analyses of self-reported family history variables found significant differences for each family history variable in all comparisons across comorbidity groups. Individuals with comorbid disorders were significantly more likely to self-report a family history of any mental health disorder than those with a single disorder, and those with anxiety-MDD were more likely than those with anxiety-anxiety comorbidity. The unadjusted models for the anxiety-MDD temporal sequence comparison found that anxiety-first anxiety-MDD was associated with a higher likelihood of self-reporting a family history of an “other” mental health disorder, but this difference was no longer significant in the partially or fully adjusted models. For the temporal sequence analyses, a follow-up analysis was run which excluded age of onset and current anxiety symptoms from the model but results still found no significant differences in family history (STable 15). Descriptives of the family history variables for the comorbidity and anxiety-MDD temporal sequence groups are displayed respectively in SFigures 5 and 6.

### Supplementary Results 4b. Genetic factors

Findings from the unadjusted analyses for the comorbidity group and temporal sequence comparisons including genetic factors are displayed respectively in STables 6 and 11, and the partially and fully adjusted model results displayed respectively in STables 16 and 17. The unadjusted model results found that the comorbidity groups were associated with significantly higher polygenic risk for ADHD and lower polygenic scores for educational attainment than the single disorders. Anxiety-MDD was also associated with significantly higher polygenic scores for neuroticism than the single disorders, and higher polygenic risk for anxiety than MDD only. The significant differences in polygenic risk between anxiety-anxiety and single anxiety were no longer significant in the partially adjusted model. In the partially adjusted analyses, anxiety-MDD remained associated with a significantly lower educational attainment PRS than single anxiety and MDD only. These differences did not remain significant in the fully adjusted models. After adjusting for all factors, anxiety-anxiety was significantly associated with higher polygenic scores for neuroticism than single anxiety. For the temporal sequence comparison, results from the unadjusted model found that individuals with anxiety-first had significantly lower polygenic risk for schizophrenia than those with MDD-first. This difference did not retain significance in the partially or fully adjusted models, even when age of onset and current anxiety symptoms were excluded from the model (STable 18). Descriptives of the polygenic risk scores for the comorbidity and anxiety-MDD temporal sequence groups are shown in SFigures 7 and 8, respectively.

# References

Chang, C.C., Chow, C.C., Tellier, L.C., Vattikuti, S., Purcell, S.M., Lee, J.J., 2015. Second-generation PLINK: rising to the challenge of larger and richer datasets. Gigascience 4, 7. doi:10.1186/s13742-015-0047-8

Coleman, J.R.I., Peyrot, W.J., Purves, K.L., Davis, K.A.S., Rayner, C., Choi, S.W., Hübel, C., Gaspar, H.A., Kan, C., Van der Auwera, S., Adams, M.J., Lyall, D.M., Choi, K.W., on the behalf of Major Depressive Disorder Working Group of the Psychiatric Genomics Consortium, Dunn, E.C., Vassos, E., Danese, A., Maughan, B., Grabe, H.J., Lewis, C.M., Breen, G., 2020. Genome-wide gene-environment analyses of major depressive disorder and reported lifetime traumatic experiences in UK Biobank. Mol. Psychiatry 25, 1430–1446. doi:10.1038/s41380-019-0546-6

Davis, K.A.S., Coleman, J.R.I., Adams, M., Allen, N., Breen, G., Cullen, B., Dickens, C., Fox, E., Graham, N., Holliday, J., Howard, L.M., John, A., Lee, W., McCabe, R., McIntosh, A., Pearsall, R., Smith, D.J., Sudlow, C., Ward, J., Zammit, S., Hotopf, M., 2020. Mental health in UK Biobank - development, implementation and results from an online questionnaire completed by 157 366 participants: a reanalysis. BJPsych Open 6, e18. doi:10.1192/bjo.2019.100

Demontis, D., Walters, R.K., Martin, J., Mattheisen, M., Als, T.D., Agerbo, E., Baldursson, G., Belliveau, R., Bybjerg-Grauholm, J., Bækvad-Hansen, M., Cerrato, F., Chambert, K., Churchhouse, C., Dumont, A., Eriksson, N., Gandal, M., Goldstein, J.I., Grasby, K.L., Grove, J., Gudmundsson, O.O., Neale, B.M., 2019. Discovery of the first genome-wide significant risk loci for attention deficit/hyperactivity disorder. Nat. Genet. 51, 63–75. doi:10.1038/s41588-018-0269-7

Ge, T., Chen, C.-Y., Ni, Y., Feng, Y.-C.A., Smoller, J.W., 2019. Polygenic prediction via Bayesian regression and continuous shrinkage priors. Nat. Commun. 10, 1776. doi:10.1038/s41467-019-09718-5

Glaesmer, H., Schulz, A., Häuser, W., Freyberger, H.J., Brähler, E., Grabe, H.-J., 2013. The Childhood Trauma Screener (CTS) - development and validation of cut-off-scores for classificatory diagnostics. Psychiatr. Prax. 40, 220–226. doi:10.1055/s-0033-1343116

Grove, J., Ripke, S., Als, T.D., Mattheisen, M., Walters, R.K., Won, H., Pallesen, J., Agerbo, E., Andreassen, O.A., Anney, R., Awashti, S., Belliveau, R., Bettella, F., Buxbaum, J.D., Bybjerg-Grauholm, J., Bækvad-Hansen, M., Cerrato, F., Chambert, K., Christensen, J.H., Churchhouse, C., Børglum, A.D., 2019. Identification of common genetic risk variants for autism spectrum disorder. Nat. Genet. 51, 431–444. doi:10.1038/s41588-019-0344-8

Kroenke, K., Spitzer, R.L., Williams, J.B.W., 2001. The PHQ-9: Validity of a brief depression severity measure. J. Gen. Intern. Med. 16, 606–613. doi:10.1046/j.1525-1497.2001.016009606.x

Lee, J.J., Wedow, R., Okbay, A., Kong, E., Maghzian, O., Zacher, M., Nguyen-Viet, T.A., Bowers, P., Sidorenko, J., Karlsson Linnér, R., Fontana, M.A., Kundu, T., Lee, C., Li, H., Li, R., Royer, R., Timshel, P.N., Walters, R.K., Willoughby, E.A., Yengo, L., Cesarini, D., 2018. Gene discovery and polygenic prediction from a genome-wide association study of educational attainment in 1.1 million individuals. Nat. Genet. 50, 1112–1121. doi:10.1038/s41588-018-0147-3

Nagel, M., Jansen, P.R., Stringer, S., Watanabe, K., de Leeuw, C.A., Bryois, J., Savage, J.E., Hammerschlag, A.R., Skene, N.G., Muñoz-Manchado, A.B., 23andMe Research Team, White, T., Tiemeier, H., Linnarsson, S., Hjerling-Leffler, J., Polderman, T.J.C., Sullivan, P.F., van der Sluis, S., Posthuma, D., 2018. Meta-analysis of genome-wide association studies for neuroticism in 449,484 individuals identifies novel genetic loci and pathways. Nat. Genet. 50, 920–927. doi:10.1038/s41588-018-0151-7

Purves, K.L., Coleman, J.R.I., Meier, S.M., Rayner, C., Davis, K.A.S., Cheesman, R., Bækvad-Hansen, M., Børglum, A.D., Wan Cho, S., Jürgen Deckert, J., Gaspar, H.A., Bybjerg-Grauholm, J., Hettema, J.M., Hotopf, M., Hougaard, D., Hübel, C., Kan, C., McIntosh, A.M., Mors, O., Bo Mortensen, P., Eley, T.C., 2020. A major role for common genetic variation in anxiety disorders. Mol. Psychiatry 25, 3292–3303. doi:10.1038/s41380-019-0559-1

Schizophrenia Working Group of the Psychiatric Genomics Consortium, 2014. Biological insights from 108 schizophrenia-associated genetic loci. Nature 511, 421–427. doi:10.1038/nature13595

Spitzer, R.L., Kroenke, K., Williams, J.B.W., Löwe, B., 2006. A brief measure for assessing generalized anxiety disorder: The GAD-7. Arch. Intern. Med. 166, 1092–1097. doi:10.1001/archinte.166.10.1092

Wray, N.R., Ripke, S., Mattheisen, M., Trzaskowski, M., Byrne, E.M., Abdellaoui, A., Adams, M.J., Agerbo, E., Air, T.M., Andlauer, T.M.F., Bacanu, S.-A., Bækvad-Hansen, M., Beekman, A.F.T., Bigdeli, T.B., Binder, E.B., Blackwood, D.R.H., Bryois, J., Buttenschøn, H.N., Bybjerg-Grauholm, J., Cai, N., et al., 2018. Genome-wide association analyses identify 44 risk variants and refine the genetic architecture of major depression. Nat. Genet. 50, 668–681. doi:10.1038/s41588-018-0090-3

# Supplementary Tables

## STable 1a. Anxiety and MDD comorbidity grouping definitions and frequencies.

| Grouping | Inclusion | Exclusion | GLAD | % GLAD | COPING NBR | % COPING NBR | Full sample | % Full sample |
| --- | --- | --- | --- | --- | --- | --- | --- | --- |
| Single anxiety | One anxiety disorder only | No MDD | 1074 | 3.05 | 450 | 12.62 | 1524 | 3.93 |
| Anxiety-anxiety | Two or more anxiety disorders | No MDD | 1489 | 4.23 | 120 | 3.37 | 1609 | 4.15 |
| Anxiety-MDD | MDD *and* one or more anxiety disorders | None | 27528 | 78.18 | 1633 | 45.81 | 29161 | 75.20 |
| MDD only | MDD diagnosis | No anxiety disorder | 5119 | 14.54 | 1362 | 38.20 | 6481 | 16.71 |

STable 1 displays the definitions and sample size by cohort and for the full sample for each of the comorbidity groups.

*Abbreviations:* MDD = major depressive disorder. GLAD = Genetic Links to Anxiety and Depression. COPING = COVID-19 Psychiatry and Neurological Genetics. NBR = NIHR BioResource

## STable 1b. Anxiety disorder frequencies by anxiety comorbidity group.

| Grouping | GAD | Specific phobia | Social anxiety disorder | Panic disorder | Agoraphobia |
| --- | --- | --- | --- | --- | --- |
| Single anxiety | 42.0% | 16.1% | 14.8% | 25.6% | 1.9% |
| Anxiety-anxiety | 76.0% | 45.0% | 56.2% | 65.0% | 34.5% |
| Anxiety-MDD | 77.1% | 30.4% | 49.8% | 56.3% | 27.0% |

STable 1b displays the frequency of symptom-based anxiety disorders for each of the anxiety comorbidity groups.

*Abbreviations:* GAD = generalised anxiety disorder. MDD = major depressive disorder.

## STable 2. Definitions for family history and self-reported diagnoses.

*Family history prompt:* Have any of your family members ever been diagnosed with one or more of the following mental health disorders by a professional, even if they don't have it currently?

*Self-reported diagnosis prompt:* Have you ever been diagnosed with the following [mental health/neurological] disorders?

| Category | Diagnoses |
| --- | --- |
| Anxiety or depressive disorder diagnosis | Depression  Depression during or after pregnancy (antenatal/postnatal depression)  Premenstrual dysphoric disorder (PMDD)  Mania, hypomania, or bipolar or manic-depression  Anxiety, nerves, or generalised anxiety disorder  Specific phobia  Social anxiety or social phobia  Agoraphobia  Panic disorder |
| Other mental health diagnosis | Post-traumatic stress disorder (PTSD)  Obsessive compulsive disorder (OCD)  Body dysmorphic disorder (BDD)  Other obsessive-compulsive related disorders (e.g., skin-picking)  Schizophrenia  Any other type of psychosis or psychotic illness  Autism spectrum disorder (ASD)  Attention deficit or attention deficit and hyperactivity disorder (ADD/ADHD)  Personality disorder  ADHD  Other  Anorexia nervosa  Atypical anorexia nervosa  Bulimia nervosa  Atypical bulimia nervosa  Binge-eating disorder  Atypical binge-eating disorder  Purging disorder  Night-eating syndrome  Pica  Avoidant/Restrictive food intake disorder (ARFID)  Rumination eating disorder  Other specified feeding or eating disorder (OSFED or EDNOS)  Other eating disorder |

STable 2 displays the response options for family history and self-reported mental health diagnoses and defines how these were categorised for analyses. The response options were the same for both measures.

*Abbreviations:* ADHD = attention deficit and hyperactivity disorder.

## STable 3a. Variance inflation factor (VIF) results for comorbidity group and temporal sequence comparisons.

| Variable | Anx-anx  (vs Anx) | Anx-MDD  (vs Anx) | Anx-anx  (vs Anx-MDD) | Anx-MDD  (vs MDD) | Anx-first  (vs MDD-first) |
| --- | --- | --- | --- | --- | --- |
| Age | 1.37 | 1.64 | 1.51 | 1.47 | 1.23 |
| Sex | 1.12 | 1.10 | 1.09 | 1.08 | 1.07 |
| Highest education | 1.13 | 1.11 | 1.11 | 1.08 | 1.09 |
| Ethnicity | 1.03 | 1.02 | 1.01 | 1.02 | 1.01 |
| Childhood trauma | 1.15 | 1.14 | 1.17 | 1.15 | 1.17 |
| Adult trauma | 1.16 | 1.13 | 1.17 | 1.14 | 1.17 |
| Catastrophic trauma | 1.16 | 1.14 | 1.18 | 1.14 | 1.18 |
| Self-reported anxiety/depressive disorder diagnosis | 1.07 | 1.06 | 1.03 | 1.03 | 1.02 |
| Other self-reported mental health diagnosis | 1.11 | 1.12 | 1.13 | 1.09 | 1.12 |
| Age of onset | 1.19 | 1.60 | 1.50 | 1.49 | 1.18 |
| Recurrence | 1.16 | 1.30 | 1.26 | 1.29 | 1.18 |
| Current depressive symptoms (PHQ9) | 1.92 | 2.18 | 2.03 | 2.04 | 2.02 |
| Current anxiety symptoms (GAD7) | 1.75 | 2.04 | 1.89 | 1.86 | 1.91 |

STable 3a displays the variance inflation factor (VIF) for all variables in the comorbidity group and temporal sequence comparisons. VIF values revealed multicollinearity between chronicity and other variables, resulting in the removal of chronicity from all further analyses. The VIF analysis for the temporal sequence comparison therefore did not include chronicity.

*Abbreviations:* Anx = Single anxiety. Anx-anx = Anxiety-anxiety. Anx-MDD = Anxiety-MDD. MDD = major depressive disorder. PHQ9 = 9-item patient health questionnaire. GAD7 = 7-item generalised anxiety disorder assessment.

## STable 3b. Variance inflation factor (VIF) results for comorbidity group comparisons, including family history variables.

| Variable | Anx-anx  (vs Anx) | Anx-MDD  (vs Anx) | Anx-anx  (vs Anx-MDD) | Anx-MDD  (vs MDD) | Anx-first  (vs MDD-first) |
| --- | --- | --- | --- | --- | --- |
| Age | 1.29 | 1.42 | 1.47 | 1.40 | 1.24 |
| Sex | 1.11 | 1.14 | 1.12 | 1.10 | 1.09 |
| Highest education | 1.13 | 1.09 | 1.10 | 1.07 | 1.08 |
| Ethnicity | 1.05 | 1.01 | 1.02 | 1.02 | 1.01 |
| Childhood trauma | 1.15 | 1.13 | 1.18 | 1.16 | 1.18 |
| Adult trauma | 1.17 | 1.15 | 1.18 | 1.15 | 1.19 |
| Catastrophic trauma | 1.23 | 1.14 | 1.20 | 1.15 | 1.19 |
| Self-reported anxiety/depressive disorder diagnosis | 1.13 | 1.11 | 1.07 | 1.07 | 1.03 |
| Other self-reported mental health diagnosis | 1.18 | 1.14 | 1.17 | 1.12 | 1.14 |
| Age of onset | 1.17 | 1.40 | 1.46 | 1.45 | 1.20 |
| Recurrence | 1.19 | 1.28 | 1.30 | 1.31 | 1.19 |
| Current depressive symptoms (PHQ9) | 2.13 | 2.21 | 2.15 | 2.05 | 2.05 |
| Current anxiety symptoms (GAD7) | 1.88 | 2.08 | 2.01 | 1.86 | 1.95 |
| Family history: Anxiety/depressive disorder | 1.20 | 1.17 | 1.15 | 1.13 | 1.13 |
| Family history: Other mental health disorder | 1.23 | 1.17 | 1.19 | 1.13 | 1.17 |

STable 3b displays the variance inflation factor (VIF) for all variables in the comorbidity group and temporal sequence comparisons including family history.

*Abbreviations:* Anx = Single anxiety. Anx-anx = Anxiety-anxiety. Anx-MDD = Anxiety-MDD. MDD = major depressive disorder. PHQ9 = 9-item patient health questionnaire. GAD7 = 7-item generalised anxiety disorder assessment.

## STable 3c. Variance inflation factor (VIF) results for comorbidity group comparisons, including genetic factors.

| Variable | Anx-anx  (vs Anx) | Anx-MDD  (vs Anx) | Anx-anx  (vs Anx-MDD) | Anx-MDD  (vs MDD) | Anx-first  (vs MDD-first) |
| --- | --- | --- | --- | --- | --- |
| Age | 1.42 | 1.56 | 1.49 | 1.42 | 1.25 |
| Sex | 1.08 | 1.09 | 1.10 | 1.09 | 1.09 |
| Highest education | 1.33 | 1.14 | 1.15 | 1.10 | 1.13 |
| Ethnicity | 1.04 | 1.01 | 1.01 | 1.01 | 1.14 |
| Childhood trauma | 1.17 | 1.14 | 1.17 | 1.14 | 1.18 |
| Adult trauma | 1.15 | 1.13 | 1.16 | 1.15 | 1.19 |
| Catastrophic trauma | 1.19 | 1.14 | 1.17 | 1.14 | 1.18 |
| Self-reported anxiety/depressive disorder diagnosis | 1.22 | 1.10 | 1.05 | 1.05 | 1.10 |
| Other self-reported mental health diagnosis | 1.14 | 1.09 | 1.12 | 1.09 | 1.13 |
| Age of onset | 1.24 | 1.52 | 1.48 | 1.47 | 1.23 |
| Recurrence | 1.20 | 1.28 | 1.28 | 1.31 | 1.23 |
| Current depressive symptoms (PHQ9) | 2.21 | 2.28 | 2.20 | 2.10 | 2.14 |
| Current anxiety symptoms (GAD7) | 1.93 | 2.13 | 2.02 | 1.90 | 1.99 |
| Anxiety PRS | 1.27 | 1.30 | 1.31 | 1.31 | 1.31 |
| MDD PRS | 1.11 | 1.06 | 1.06 | 1.05 | 1.06 |
| Neuroticism PRS | 1.32 | 1.26 | 1.29 | 1.29 | 1.28 |
| ADHD PRS | 1.21 | 1.16 | 1.17 | 1.16 | 1.17 |
| Autism PRS | 1.24 | 1.15 | 1.16 | 1.15 | 1.15 |
| Schizophrenia PRS | 1.26 | 1.19 | 1.19 | 1.19 | 1.21 |
| Educational attainment PRS | 1.16 | 1.13 | 1.14 | 1.16 | 1.17 |
| Genotyping batch | 4.62 | 2.78 | 2.22 | 2.08 | 1.58 |
| Principal component 1 | 1.17 | 1.14 | 1.11 | 1.11 | 1.16 |
| Principal component 2 | 1.08 | 1.04 | 1.05 | 1.03 | 1.05 |
| Principal component 3 | 1.12 | 1.05 | 1.03 | 1.03 | 1.04 |
| Principal component 4 | 1.23 | 1.16 | 1.25 | 1.22 | 1.62 |
| Principal component 5 | 1.17 | 1.04 | 1.18 | 1.10 | 1.54 |
| Principal component 6 | 1.12 | 1.08 | 1.13 | 1.18 | 1.39 |
| Principal component 7 | 1.32 | 1.25 | 1.24 | 1.27 | 1.42 |
| Principal component 8 | 1.10 | 1.07 | 1.09 | 1.08 | 1.14 |
| Principal component 9 | 1.26 | 1.13 | 1.18 | 1.15 | 1.22 |
| Principal component 10 | 1.13 | 1.09 | 1.06 | 1.06 | 1.08 |

STable 3c displays the variance inflation factor (VIF) for all variables in the comorbidity group and temporal sequence comparisons including genetic factors.

*Abbreviations:* Anx = Single anxiety. Anx-anx = Anxiety-anxiety. Anx-MDD = Anxiety-MDD. MDD = major depressive disorder. PRS = polygenic risk score. ADHD = attention deficit and hyperactivity disorder. PHQ9 = 9-item patient health questionnaire. GAD7 = 7-item generalised anxiety disorder assessment.

## STable 4. Descriptives for full sample and by cohort.

| Variable | | GLAD | COPING NBR | Full sample |
| --- | --- | --- | --- | --- |
| Sociodemographic | |  |  |  |
|  | Age (years, sd) | 37.50 (14.20) | 50.70 (14.00) | 38.70 (14.70) |
|  | Sex (% female, n) | 81.1 (28552) | 69.9 (2492) | 80.1 (31044) |
|  | Ethnicity (% white, n) | 94.6 (33235) | 96.7 (2828) | 94.8 (36063) |
|  | Highest education: University degree (%, n) | 52.8 (18345) | 52.3 (1831) | 52.7 (20176) |
|  | Highest education: A-levels (%, n) | 23.6 (8184) | 21.2 (743) | 23.3 (8927) |
|  | Highest education: GCSEs (%, n) | 21.2 (7357) | 24.1 (844) | 21.4 (8201) |
|  | Highest education: No qualifications (%, n) | 2.5 (864) | 2.3 (82) | 2.5 (946) |
| Trauma | |  |  |  |
|  | Childhood trauma (% present, n) | 58 (20127) | 38.5 (1343) | 56.2 (21470) |
|  | Adult trauma (% present, n) | 46.4 (15662) | 27.9 (949) | 44.7 (16611) |
|  | Catastrophic trauma (% present, n) | 67.9 (22821) | 60.1 (2047) | 67.2 (24868) |
| Clinical | |  |  |  |
|  | Self-reported anxiety/depressive disorder  (% diagnosis, n) | 97.1 (34122) | 63.4 (2249) | 94 (36371) |
|  | Self-reported other mental health disorder  (% diagnosis, n) | 44.1 (15502) | 16.1 (570) | 41.5 (16072) |
|  | Age of onset (years, sd) | 15.90 (9.50) | 24.40 (14.60) | 16.70 (10.40) |
|  | Chronicity (years, sd) | 9.30 (4.00) | 6.00 (4.70) | 8.90 (4.20) |
|  | Recurrence (number of episodes, sd) | 12.70 (7.00) | 6.50 (5.80) | 12.10 (7.10) |
|  | Current depression (total score, sd) | 10.20 (6.10) | 5.20 (5.20) | 9.70 (6.20) |
|  | Current anxiety (total score, sd) | 97.1 (34122) | 63.4 (2249) | 94.0 (36371) |
| Self-reported family history | |  |  |  |
|  | Family history: Anxiety/depressive disorder (% positive, n) | 74.9 (11040) | 54.8 (1950) | 71 (12990) |
|  | Family history: Other mental health disorder (% positive, n) | 42.1 (6050) | 24.1 (856) | 38.5 (6906) |
| Genetic | |  |  |  |
|  | Anxiety PRS (total score, sd) | 0.06 (1.00) | 0.01 (0.99) | 0.06 (1.00) |
|  | MDD PRS (total score, sd) | 0.04 (1.00) | -0.02 (1.00) | 0.03 (1.00) |
|  | Neuroticism PRS (total score, sd) | 0.07 (1.00) | 0.01 (0.96) | 0.06 (1.00) |
|  | ADHD PRS (total score, sd) | 0.04 (1.00) | -0.04 (0.99) | 0.03 (1.00) |
|  | Autism PRS (total score, sd) | 0.04 (1.00) | 0.02 (1.00) | 0.04 (1.00) |
|  | Schizophrenia PRS (total score, sd) | 0.06 (0.99) | 0.01 (1.00) | 0.06 (0.99) |
|  | Educational attainment PRS (total score, sd) | -0.04 (1.00) | 0.05 (0.98) | -0.03 (1.00) |

STable 4 displays the descriptives and frequencies for each of the variables included in the analyses for the full sample and by cohort. Current depression and anxiety scores were measured using the PHQ-9 and GAD-7 and calculated by summing the responses for each item in the scale.

*Abbreviations:* GLAD = Genetic Links to Anxiety and Depression. COPING = COVID-19 Psychiatry and Neurological Genetics. NBR = NIHR BioResource. sd = standard deviation. N = sample size. PRS = polygenic risk score. MDD = major depressive disorder. PHQ9 = 9-item patient health questionnaire. GAD7 = 7-item generalised anxiety disorder assessment.

## STable 5. Sample size for each model.

|  | Partially adjusted models | | | | | Fully adjusted models | | |
| --- | --- | --- | --- | --- | --- | --- | --- | --- |
| Outcome | Socio-  demographic | Trauma | Clinical | Family history | Genetic | Main analysis | Including family history | Including genetics |
| Anxiety-anxiety  (vs single anxiety) | 2991 | 2927 | 1982 | 1528 | 1297 | 1752 | 847 | 662 |
| Anxiety-MDD  (vs single anxiety) | 29840 | 28413 | 26045 | 13525 | 12525 | 23427 | 10332 | 9344 |
| Anxiety-anxiety  (vs anxiety-MDD) | 29995 | 28480 | 26467 | 13383 | 12470 | 23839 | 10383 | 9416 |
| Anxiety-MDD  (vs MDD only) | 34551 | 33197 | 30702 | 16318 | 14842 | 27652 | 12640 | 11287 |
| Temporal sequence | 18221 | 17340 | 16464 | 8186 | 7472 | 14875 | 6523 | 5785 |

STable 5 displays the sample size included in the analyses specified in the row headers, for each of the comparisons detailed in the “Outcome” column.

*Abbreviations:* MDD = major depressive disorder.

## STable 6. Results from unadjusted logistic regression models for the comorbidity group comparisons.

|  |  | Unadjusted analyses OR (95% CI) | | |  | |
| --- | --- | --- | --- | --- | --- | --- |
| Variable | Anx-anx (vs Anx) | | Anx-MDD (vs Anx) | Anx-anx (vs Anx-MDD) | | Anx-MDD (vs MDD only) |
| Age | 0.77*** (0.74, 0.81) | | 0.82*** (0.79, 0.85) | 0.91*** (0.88, 0.94) | | 0.79*** (0.78, 0.81) |
| Female | 1.29** (1.09, 1.52) | | 1.63*** (1.45, 1.83) | 0.79*** (0.70, 0.89) | | 1.56*** (1.47, 1.66) |
| GCSEs or equivalent | 1.08 (0.68, 1.71) | | 0.99 (0.68, 1.39) | 1.09 (0.81, 1.51) | | 0.69** (0.55, 0.86) |
| A-levels or equivalent | 0.88 (0.56, 1.40) | | 1.06 (0.73, 1.48) | 0.84 (0.62, 1.16) | | 0.68*** (0.54, 0.84) |
| University degree | 0.68 (0.44, 1.06) | | 0.80 (0.56, 1.11) | 0.85 (0.64, 1.17) | | 0.46*** (0.37, 0.57) |
| White | 0.98 (0.70, 1.36) | | 0.94 (0.73, 1.19) | 1.04 (0.83, 1.32) | | 1.04 (0.92, 1.17) |
| Childhood trauma | 1.53*** (1.33, 1.77) | | 2.29*** (2.06, 2.55) | 0.67*** (0.60, 0.74) | | 2.05*** (1.94, 2.17) |
| Adult trauma | 1.79*** (1.53, 2.09) | | 2.75*** (2.44, 3.10) | 0.65*** (0.59, 0.72) | | 1.94*** (1.83, 2.06) |
| Catastrophic trauma | 1.23** (1.07, 1.42) | | 2.09*** (1.88, 2.32) | 0.59*** (0.53, 0.66) | | 1.51*** (1.42, 1.60) |
| Self-reported anxiety/depressive disorder diagnosis | 3.41*** (2.77, 4.21) | | 9.40*** (8.22, 10.73) | 0.36*** (0.30, 0.44) | | 4.23*** (3.84, 4.66) |
| Other self-reported mental health diagnosis | 1.88*** (1.61, 2.19) | | 2.59*** (2.30, 2.92) | 0.73*** (0.65, 0.80) | | 2.53*** (2.38, 2.69) |
| Age of onset | 0.73*** (0.68, 0.77) | | 0.56*** (0.53, 0.59) | 1.24*** (1.18, 1.30) | | 0.48*** (0.47, 0.50) |
| Recurrence | 1.12*** (1.10, 1.14) | | 1.16*** (1.15, 1.18) | 0.98** (0.97, 1.00) | | 1.20*** (1.19, 1.21) |
| Current depressive symptoms (PHQ9) | 1.08*** (1.07, 1.10) | | 1.12*** (1.11, 1.13) | 0.96*** (0.96, 0.97) | | 1.11*** (1.11, 1.12) |
| Current anxiety symptoms (GAD7) | 1.11*** (1.10, 1.13) | | 1.10*** (1.09, 1.11) | 1.01*** (1.01, 1.02) | | 1.18*** (1.17, 1.19) |
| Family history: Anxiety/depressive disorder | 1.36** (1.11, 1.68) | | 1.83*** (1.59, 2.11) | 0.74*** (0.63, 0.88) | | 1.56*** (1.44, 1.68) |
| Family history: Other mental health disorder | 1.37** (1.10, 1.70) | | 1.75*** (1.50, 2.04) | 0.78** (0.67, 0.92) | | 1.70*** (1.57, 1.84) |
| Anxiety PRS | 1.02 (0.92, 1.14) | | 1.00 (0.93, 1.08) | 1.02 (0.94, 1.11) | | 1.06** (1.02, 1.11) |
| MDD PRS | 1.04 (0.93, 1.15) | | 1.02 (0.94, 1.10) | 1.02 (0.94, 1.10) | | 1.03 (0.99, 1.08) |
| Neuroticism PRS | 1.10 (0.98, 1.23) | | 1.09* (1.01, 1.18) | 1.00 (0.92, 1.08) | | 1.08*** (1.04, 1.13) |
| ADHD PRS | 1.14* (1.02, 1.26) | | 1.10* (1.02, 1.19) | 1.04 (0.96, 1.13) | | 1.06** (1.02, 1.10) |
| Autism PRS | 1.07 (0.96, 1.20) | | 1.00 (0.93, 1.08) | 1.06 (0.98, 1.15) | | 1.01 (0.97, 1.05) |
| Schizophrenia PRS | 0.98 (0.88, 1.09) | | 1.01 (0.93, 1.09) | 0.97 (0.89, 1.05) | | 1.03 (0.99, 1.07) |
| Educational attainment PRS | 0.83*** (0.74, 0.93) | | 0.85*** (0.79, 0.92) | 0.97 (0.89, 1.05) | | 0.90*** (0.87, 0.94) |

STable 6. Results from unadjusted logistic regression models for the comorbidity group comparisons. The table displays the odds ratios and confidence intervals for the unadjusted logistic regression models, in which each variable was entered separately into a univariate logistic regression model for each comparison indicated in the column headers. The column headers also indicate the direction of the comparison, with the reference group in parentheses. For example, in the first column “Anx-anx (vs Anx)”, anxiety-anxiety comorbidity is being compared to single anxiety, such that OR > 1 indicates a higher likelihood of having anxiety-anxiety. The significance values (q) were adjusted using the false discovery rate multiple testing correction. Note that confidence intervals, while reported, are not fully informative due to the adjustment for multiple testing.

*Abbreviations:* Anx = Single anxiety; Anx-anx = Anxiety-anxiety; Anx-MDD = Anxiety-MDD; OR = odds ratio; CI = confidence interval; PHQ9 = 9-item patient health questionnaire; GAD7 = 7-item generalised anxiety disorder assessment

*q < 0.05, **q < 0.01, ***q < 0.001

## STable 7. Results from the fully adjusted logistic regression model for anxiety-anxiety vs single anxiety and anxiety-MDD vs MDD only, excluding current anxiety symptoms.

| Variable | | Anx-Anx (vs Anx)  OR (95% CI) | Anx-MDD (vs MDD only)  OR (95% CI) |
| --- | --- | --- | --- |
| Sociodemographic | |  |  |
|  | Age | 0.87** (0.80, 0.94) | 1.00 (0.97, 1.03) |
|  | Female | 1.20 (0.93, 1.56) | 1.08 (0.99, 1.18) |
|  | University degree | 0.64 (0.31, 1.27) | 0.61*** (0.45, 0.80) |
|  | A-levels or equivalent | 0.60 (0.28, 1.21) | 0.69* (0.51, 0.92) |
|  | GCSEs or equivalent | 0.76 (0.36, 1.51) | 0.76 (0.56, 1.01) |
|  | White | 1.12 (0.66, 1.87) | 1.28** (1.10, 1.49) |
| Vulnerability | |  |  |
|  | Childhood trauma | 1.08 (0.86, 1.36) | 1.12** (1.04, 1.20) |
|  | Adult trauma | 1.24 (0.97, 1.59) | 1.27*** (1.18, 1.37) |
|  | Catastrophic trauma | 0.99 (0.78, 1.25) | 1.00 (0.92, 1.08) |
| Clinical | |  |  |
|  | Self-reported anxiety/depressive disorder diagnosis | 3.11*** (2.31, 4.22) | 2.09*** (1.83, 2.39) |
|  | Other self-reported mental health diagnosis | 1.08 (0.85, 1.38) | 1.37*** (1.27, 1.48) |
|  | Age of onset | 0.76*** (0.70, 0.83) | 0.61*** (0.59, 0.63) |
|  | Recurrence | 1.08*** (1.05, 1.10) | 1.08*** (1.07, 1.09) |
|  | Current depressive symptoms (PHQ9) | 1.04*** (1.02, 1.07) | 1.05*** (1.05, 1.06) |

STable 7. Results from the fully adjusted logistic regression model for the comparison between anxiety-anxiety vs single anxiety and anxiety-MDD vs MDD only, excluding current anxiety symptoms. The table displays the odds ratios and confidence intervals for the fully adjusted logistic regression model comparing anxiety-anxiety comorbidity and single anxiety without current anxiety symptoms (GAD7). The model included all variables. The significance values (q) were adjusted using the false discovery rate multiple testing correction. Note that confidence intervals, while reported, are not fully informative due to the adjustment for multiple testing.

*Abbreviations:* Anx-anx = Anxiety-anxiety. Anx = Single anxiety. OR = odds ratio. CI = confidence interval. PHQ9 = 9-item patient health questionnaire. GAD7 = 7-item generalised anxiety disorder assessment.

*q < 0.05, **q < 0.01, ***q < 0.001

## STable 8. Results from the fully adjusted logistic regression model for anxiety-anxiety vs anxiety-MDD, excluding age of onset and current depressive symptoms.

| Variable | | Anx-Anx (vs Anx-MDD)  OR (95% CI) |
| --- | --- | --- |
| Sociodemographic | |  |
|  | Age | 0.93** (0.89, 0.97) |
|  | Female | 0.85* (0.74, 0.99) |
|  | University degree | 0.69* (0.49, 0.99) |
|  | A-levels or equivalent | 0.66* (0.46, 0.96) |
|  | GCSEs or equivalent | 0.97 (0.69, 1.41) |
|  | White | 1.10 (0.84, 1.48) |
| Vulnerability | |  |
|  | Childhood trauma | 0.81** (0.71, 0.92) |
|  | Adult trauma | 0.78*** (0.68, 0.88) |
|  | Catastrophic trauma | 0.73*** (0.64, 0.83) |
| Clinical | |  |
|  | Self-reported anxiety/depressive disorder diagnosis | 0.45*** (0.36, 0.57) |
|  | Other self-reported mental health diagnosis | 0.81** (0.71, 0.92) |
|  | Recurrence | 0.99 (0.98, 1.01) |
|  | Current anxiety symptoms (GAD7) | 1.02*** (1.01, 1.03) |

STable 8. Results from the fully adjusted logistic regression model for the comparison between anxiety-anxiety vs anxiety-MDD, excluding age of onset and current depressive symptoms. The table displays the odds ratios and confidence intervals for the fully adjusted logistic regression model comparing anxiety-anxiety and anxiety-MDD comorbidity without age of onset and current depressive symptoms. The model included all listed variables. The significance values (q) were adjusted using the false discovery rate multiple testing correction. Note that confidence intervals, while reported, are not fully informative due to the adjustment for multiple testing.

*Abbreviations:* Anx-anx = Anxiety-anxiety. Anx-MDD = Anxiety-MDD. MDD = major depressive disorder. OR = odds ratio. CI = confidence interval. PHQ9 = 9-item patient health questionnaire. GAD7 = 7-item generalised anxiety disorder assessment.

*q < 0.05, **q < 0.01, ***q < 0.001

## STable 9. Results from the fully adjusted logistic regression model for anxiety-MDD vs MDD only, excluding age of onset.

| Variable | | Anx-MDD (vs MDD only)  OR (95% CI) |
| --- | --- | --- |
| Sociodemographic | |  |
|  | Age | 0.91*** (0.89, 0.93) |
|  | Female | 1.23*** (1.13, 1.34) |
|  | University degree | 0.75 (0.56, 0.99) |
|  | A-levels or equivalent | 0.86 (0.64, 1.15) |
|  | GCSEs or equivalent | 0.86 (0.64, 1.14) |
|  | White | 1.27** (1.09, 1.48) |
| Vulnerability | |  |
|  | Childhood trauma | 1.21*** (1.13, 1.30) |
|  | Adult trauma | 1.28*** (1.18, 1.38) |
|  | Catastrophic trauma | 1.07 (1.00, 1.16) |
| Clinical | |  |
|  | Self-reported anxiety/depressive disorder diagnosis | 2.21*** (1.94, 2.52) |
|  | Other self-reported mental health diagnosis | 1.43*** (1.33, 1.55) |
|  | Recurrence | 1.11*** (1.10, 1.12) |
|  | Current depressive symptoms (PHQ9) | 0.97*** (0.97, 0.98) |
|  | Current anxiety symptoms (GAD7) | 1.15*** (1.14, 1.16) |

STable 9. Results from the fully adjusted logistic regression model for the comparison between anxiety-MDD vs MDD only, excluding age of onset. The table displays the odds ratios and confidence intervals for the fully adjusted logistic regression model comparing anxiety-anxiety and anxiety-MDD comorbidity without age of onset. The model included all variables. The significance values (q) were adjusted using the false discovery rate multiple testing correction. Note that confidence intervals, while reported, are not fully informative due to the adjustment for multiple testing.

*Abbreviations:* Anx-anx = Anxiety-anxiety. Anx-MDD = Anxiety-MDD. MDD = major depressive disorder. OR = odds ratio. CI = confidence interval. PHQ9 = 9-item patient health questionnaire. GAD7 = 7-item generalised anxiety disorder assessment.

*q < 0.05, **q < 0.01, ***q < 0.001

## STable 10. Results from partially adjusted and fully adjusted logistic regression models for comorbidity group comparisons, excluding self-reported anxiety or depressive disorder diagnosis.

|  | |  |  | Partially adjusted models | |  |  | Fully adjusted models | |  |
| --- | --- | --- | --- | --- | --- | --- | --- | --- | --- | --- |
| Variable | | | Anx-anx  (vs Anx)  OR (95% CI) | Anx-MDD  (vs Anx)  OR (95% CI) | Anx-anx  (vs Anx-MDD)  OR (95% CI) | Anx-MDD  (vs MDD only)  OR (95% CI) | Anx-anx  (vs Anx)  OR (95% CI) | Anx-MDD  (vs Anx)  OR (95% CI) | Anx-anx  (vs Anx-MDD)  OR (95% CI) | Anx-MDD  (vs MDD only)  OR (95% CI) |
| Sociodemographic | | |  |  |  |  |  |  |  |  |
|  | Age | | 0.77***  (0.73, 0.81) | 0.84***  (0.81, 0.87) | 0.89***  (0.85, 0.92) | 0.80***  (0.78, 0.82) | 0.87***  (0.80, 0.94) | 1.03  (0.96, 1.10) | 0.81***  (0.77, 0.85) | 1.05***  (1.02, 1.08) |
|  | Female | | 1.14  (0.95, 1.35) | 1.45***  (1.28, 1.64) | 0.74***  (0.66, 0.84) | 1.37***  (1.28, 1.47) | 1.18  (0.91, 1.53) | 1.16  (0.96, 1.39) | 0.92  (0.78, 1.07) | 1.10*  (1.01, 1.20) |
|  | University degree | | 0.51**  (0.32, 0.81) | 0.71  (0.49, 0.99) | 0.84  (0.63, 1.15) | 0.39***  (0.31, 0.49) | 0.82  (0.40, 1.62) | 0.99  (0.55, 1.68) | 0.93  (0.65, 1.36) | 0.83  (0.61, 1.10) |
|  | A-levels or equivalent | | 0.61  (0.37, 0.98) | 0.87  (0.59, 1.23) | 0.77  (0.57, 1.07) | 0.53***  (0.42, 0.66) | 0.66  (0.32, 1.33) | 1.03  (0.57, 1.76) | 0.62*  (0.43, 0.91) | 0.79  (0.58, 1.05) |
|  | GCSEs or equivalent | | 0.90  (0.56, 1.46) | 0.95  (0.65, 1.34) | 1.08  (0.80, 1.50) | 0.64***  (0.50, 0.80) | 0.72  (0.35, 1.41) | 1.16  (0.65, 1.93) | 0.59**  (0.42, 0.86) | 0.69*  (0.51, 0.91) |
|  | White | | 1.09  (0.78, 1.53) | 1.03  (0.79, 1.30) | 1.07  (0.86, 1.37) | 1.17**  (1.03, 1.33) | 1.41  (0.84, 2.33) | 1.04  (0.69, 1.52) | 1.05  (0.79, 1.42) | 1.31**  (1.12, 1.52) |
| Trauma | | |  |  |  |  |  |  |  |  |
|  | Childhood trauma | | 1.39***  (1.19, 1.62) | 1.78***  (1.59, 2.00) | 0.78***  (0.70, 0.87) | 1.79***  (1.69, 1.90) | 1.13  (0.90, 1.42) | 1.19  (1.00, 1.41) | 0.92  (0.80, 1.05) | 1.12**  (1.04, 1.20) |
|  | Adult trauma | | 1.63***  (1.38, 1.92) | 2.16***  (1.91, 2.45) | 0.75***  (0.67, 0.84) | 1.67***  (1.57, 1.77) | 1.32*  (1.04, 1.70) | 1.62***  (1.35, 1.96) | 0.78**  (0.68, 0.90) | 1.25***  (1.16, 1.35) |
|  | Catastrophic trauma | | 1.01  (0.87, 1.19) | 1.45***  (1.30, 1.63) | 0.69***  (0.62, 0.78) | 1.11***  (1.04, 1.18) | 1.02  (0.81, 1.29) | 1.31**  (1.10, 1.55) | 0.77***  (0.67, 0.89) | 1.02  (0.95, 1.10) |
| Clinical | | |  |  |  |  |  |  |  |  |
|  | Other self-reported mental health diagnosis | | 1.42**  (1.14, 1.77) | 1.69***  (1.42, 2.01) | 0.82**  (0.72, 0.92) | 1.44***  (1.34, 1.55) | 1.20  (0.94, 1.53) | 1.41***  (1.17, 1.70) | 0.84*  (0.74, 0.96) | 1.37***  (1.27, 1.48) |
|  | Age of onset | | 0.74***  (0.69, 0.80) | 0.61***  (0.58, 0.65) | 1.27***  (1.20, 1.34) | 0.62***  (0.60, 0.64) | 0.79***  (0.73, 0.86) | 0.61***  (0.57, 0.66) | 1.41***  (1.31, 1.51) | 0.60***  (0.58, 0.63) |
|  | Recurrence | | 1.07***  (1.04, 1.09) | 1.04***  (1.02, 1.06) | 1.02**  (1.01, 1.04) | 1.09***  (1.08, 1.10) | 1.08***  (1.05, 1.10) | 1.04***  (1.02, 1.06) | 1.04***  (1.02, 1.05) | 1.08***  (1.07, 1.09) |
|  | Current depressive symptoms (PHQ9) | | 1.02*  (1.00, 1.05) | 1.09***  (1.07, 1.11) | 0.93***  (0.92, 0.94) | 0.98***  (0.97, 0.99) | 1.01  (0.98, 1.03) | 1.08***  (1.06, 1.10) | 0.92***  (0.91, 0.94) | 0.98***  (0.97, 0.98) |
|  | Current anxiety symptoms (GAD7) | | 1.09***  (1.06, 1.11) | 1.00  (0.98, 1.02) | 1.08***  (1.07, 1.10) | 1.15***  (1.14, 1.16) | 1.08***  (1.06, 1.11) | 1.00  (0.98, 1.02) | 1.08***  (1.06, 1.09) | 1.15***  (1.14, 1.17) |

STable 10. Results from partially adjusted and fully adjusted logistic regression models for comorbidity group comparisons, excluding self-reported anxiety or depressive disorder diagnosis. The table displays the odds ratios and confidence intervals for the sensitivity analysis excluding the self-reported anxiety or depressive disorder diagnosis explanatory variable, with the logistic regression models for each comparison indicated in the column headers. The column headers also indicate the direction of the comparison, with the reference group in parentheses. For example, in the first column “Anx-anx (vs Anx)”, anxiety-anxiety comorbidity is being compared to single anxiety, such that OR > 1 indicates a higher likelihood of having anxiety-anxiety. The partially adjusted models refer to the regressions run with each group of independent variables separately (e.g., sociodemographic variables only). The full models include all three sets of variables. The significance values (q) were adjusted using the false discovery rate multiple testing correction. Note that confidence intervals, while reported, are not fully informative due to the adjustment for multiple testing.

Abbreviations: Anx = Single anxiety; Anx-anx = Anxiety-anxiety; Anx-MDD = Anxiety-MDD; OR = odds ratio; CI = confidence interval;

PHQ9 = 9-item patient health questionnaire; GAD7 = 7-item generalised anxiety disorder assessment

*q < 0.05, **q < 0.01, ***q < 0.001

## STable 11. Results from the unadjusted logistic regression models for temporal sequence comparison.

|  | Unadjusted analyses  OR (95% CI) |
| --- | --- |
| Variable | Anxiety-first (vs MDD-first) |
| Age | 0.97** (0.95, 0.99) |
| Female | 1.43*** (1.33, 1.55) |
| GCSEs or equivalent | 0.83 (0.68, 1.01) |
| A-levels or equivalent | 0.80* (0.65, 0.97) |
| University degree | 0.71** (0.59, 0.86) |
| White | 1.15* (1.01, 1.31) |
| Childhood trauma | 1.11*** (1.05, 1.18) |
| Adult trauma | 1.03 (0.97, 1.09) |
| Catastrophic trauma | 1.04 (0.97, 1.10) |
| Self-reported anxiety/depressive disorder diagnosis | 0.86 (0.73, 1.00) |
| Other self-reported mental health diagnosis | 1.09** (1.02, 1.15) |
| Age of onset | 0.16*** (0.15, 0.17) |
| Recurrence | 1.03*** (1.02, 1.04) |
| Current depressive symptoms (PHQ9) | 1.01** (1.00, 1.01) |
| Current anxiety symptoms (GAD7) | 1.03*** (1.03, 1.04) |
| Family history: Anxiety/depressive disorder | 1.10 (1.00, 1.21) |
| Family history: Other mental health disorder | 1.11* (1.02, 1.22) |
| Anxiety PRS | 0.97 (0.93, 1.01) |
| MDD PRS | 1.02 (0.97, 1.06) |
| Neuroticism PRS | 0.98 (0.94, 1.03) |
| ADHD PRS | 1.01 (0.97, 1.06) |
| Autism PRS | 0.98 (0.94, 1.03) |
| Schizophrenia PRS | 0.95* (0.91, 1.00) |
| Educational attainment PRS | 0.97 (0.93, 1.01) |

STable 11. Results from the unadjusted logistic regression models for temporal sequence analyses. The table displays the odds ratios and confidence intervals for the unadjusted logistic regression models, in which each variable was entered separately into a univariate logistic regression model for anxiety-first compared to MDD-first. The significance values (q) were adjusted using the false discovery rate multiple testing correction. Note that confidence intervals, while reported, are not fully informative due to the adjustment for multiple testing.

*Abbreviations:* OR = odds ratio. CI = confidence interval. PHQ9 = 9-item patient health questionnaire. GAD7 = 7-item generalised anxiety disorder assessment.

*q < 0.05, **q < 0.01, ***q < 0.001

## STable 12. Results from partially adjusted and fully adjusted logistic regression models for temporal sequence comparison, excluding age of onset and current anxiety symptoms.

|  | |  | Anxiety-first (vs MDD-first) | |
| --- | --- | --- | --- | --- |
| Variable | | | Partially adjusted model  OR (95% CI) | Fully adjusted model  OR (95% CI) |
| Sociodemographic | | |  |  |
|  | Age | | 0.98 (0.96, 1.00) | 0.99 (0.97, 1.01) |
|  | Female | | 1.44*** (1.33, 1.56) | 1.46*** (1.33, 1.60) |
|  | University degree | | 0.69*** (0.57, 0.84) | 0.84 (0.67, 1.06) |
|  | A-levels or equivalent | | 0.76* (0.62, 0.93) | 0.81 (0.65, 1.02) |
|  | GCSEs or equivalent | | 0.82 (0.67, 0.99) | 0.74* (0.59, 0.92) |
|  | White | | 1.15 (1.01, 1.31) | 1.14 (0.98, 1.31) |
| Trauma | | |  |  |
|  | Childhood trauma | | 1.10** (1.04, 1.18) | 1.06 (0.99, 1.14) |
|  | Adult trauma | | 1.01 (0.95, 1.08) | 0.93 (0.87, 1.00) |
|  | Catastrophic trauma | | 1.00 (0.93, 1.07) | 1.02 (0.94, 1.10) |
| Clinical | | |  |  |
|  | Self-reported anxiety/depressive disorder diagnosis | | 0.79* (0.67, 0.94) | 0.77* (0.64, 0.93) |
|  | Other self-reported mental health diagnosis | | 1.05 (0.98, 1.12) | 1.03 (0.96, 1.11) |
|  | Recurrence | | 1.03*** (1.02, 1.03) | 1.03*** (1.02, 1.03) |
|  | Current depressive symptoms (PHQ9) | | 1.00 (1.00, 1.01) | 1.00 (1.00, 1.01) |

STable 12. Results from partially adjusted and fully adjusted logistic regression models for temporal sequence analyses excluding age of onset and current anxiety symptoms. The table displays the odds ratios and confidence intervals for the logistic regression models for each comparison indicated in the column headers. The partially adjusted models refer to the regressions run with each group of independent variables separately (e.g., sociodemographic variables only). The clinical model is the only partially adjusted analysis with different results from the model in the main manuscript, as it now excludes age of onset and current anxiety symptoms (GAD7). The full model includes all listed variables. The significance values (q) were adjusted using the false discovery rate multiple testing correction. Note that confidence intervals, while reported, are not fully informative due to the adjustment for multiple testing.

*Abbreviations:* OR = odds ratio. CI = confidence interval. PHQ9 = 9-item patient health questionnaire. GAD7 = 7-item generalised anxiety disorder assessment.

*q < 0.05, **q < 0.01, ***q < 0.001

## STable 13. Results from partially adjusted and fully adjusted logistic regression models for comorbidity group comparisons, including self-reported family history.

|  | |  |  | Partially adjusted models | |  |  | Fully adjusted models | |  |  |
| --- | --- | --- | --- | --- | --- | --- | --- | --- | --- | --- | --- |
| Variable | | | Anx-anx  (vs Anx)  OR (95% CI) | Anx-MDD  (vs Anx)  OR (95% CI) | Anx-anx  (vs Anx-MDD)  OR (95% CI) | Anx-MDD  (vs MDD only)  OR (95% CI) | Anx-anx  (vs Anx)  OR (95% CI) | Anx-MDD  (vs Anx)  OR (95% CI) | Anx-anx  (vs Anx-MDD)  OR (95% CI) | Anx-MDD  (vs MDD only)  OR (95% CI) |  |
| Sociodemographic | | |  |  |  |  |  |  |  |  |  |
|  | Age | | 0.77***  (0.73, 0.81) | 0.84***  (0.81, 0.87) | 0.89***  (0.85, 0.92) | 0.80***  (0.78, 0.82) | 0.82**  (0.73, 0.93) | 0.94  (0.86, 1.03) | 0.88**  (0.81, 0.95) | 1.02  (0.98, 1.07) |  |
|  | Female | | 1.14  (0.95, 1.35) | 1.45***  (1.28, 1.64) | 0.74***  (0.66, 0.84) | 1.37***  (1.28, 1.47) | 1.41  (0.96, 2.09) | 1.25  (0.95, 1.62) | 0.96  (0.75, 1.25) | 1.07  (0.95, 1.22) |  |
|  | University degree | | 0.51**  (0.32, 0.81) | 0.71  (0.49, 0.99) | 0.84  (0.63, 1.15) | 0.39***  (0.31, 0.49) | 0.98  (0.34, 2.76) | 1.19  (0.51, 2.54) | 0.82  (0.46, 1.58) | 0.93  (0.58, 1.45) |  |
|  | A-levels or equivalent | | 0.61  (0.37, 0.98) | 0.87  (0.59, 1.23) | 0.77  (0.57, 1.07) | 0.53***  (0.42, 0.66) | 0.79  (0.27, 2.26) | 1.18  (0.50, 2.53) | 0.59  (0.33, 1.13) | 0.91  (0.57, 1.42) |  |
|  | GCSEs or equivalent | | 0.90  (0.56, 1.46) | 0.95  (0.65, 1.34) | 1.08  (0.80, 1.50) | 0.64***  (0.50, 0.80) | 0.75  (0.26, 2.07) | 1.27  (0.55, 2.65) | 0.53  (0.30, 1.01) | 0.75  (0.47, 1.15) |  |
|  | White | | 1.09  (0.78, 1.53) | 1.03  (0.79, 1.30) | 1.07  (0.86, 1.37) | 1.17**  ( 1.03, 1.33) | 0.89  (0.35, 2.11) | 0.72  (0.33, 1.38) | 0.81  (0.53, 1.32) | 1.17  (0.92, 1.49) |  |
| Trauma | | |  |  |  |  |  |  |  |  |  |
|  | Childhood trauma | | 1.39***  (1.19, 1.62) | 1.78***  (1.59, 2.00) | 0.78***  (0.70, 0.87) | 1.79***  (1.69, 1.90) | 1.07  (0.76, 1.51) | 1.31  (1.03, 1.66) | 0.78  (0.63, 0.96) | 1.13  (1.02, 1.26) |  |
|  | Adult trauma | | 1.63***  (1.38, 1.92) | 2.16***  (1.91, 2.45) | 0.75***  (0.67, 0.84) | 1.67***  (1.57, 1.77) | 1.12  (0.78, 1.61) | 1.29  (0.99, 1.67) | 0.82  (0.66, 1.01) | 1.20**  (1.08, 1.34) |  |
|  | Catastrophic trauma | | 1.01 (0.87, 1.19) | 1.45***  (1.30, 1.63) | 0.69***  (0.62, 0.78) | 1.11***  (1.04, 1.18) | 0.83  (0.58, 1.17) | 1.30  (1.03, 1.65) | 0.71**  (0.58, 0.88) | 1.05  (0.94, 1.17) |  |
| Clinical | | |  |  |  |  |  |  |  |  |  |
|  | Self-reported anxiety/depressive disorder diagnosis | | 3.10***  (2.35, 4.10) | 7.98***  (6.63, 9.58) | 0.39***  (0.32, 0.49) | 2.20***  (1.94, 2.48) | 3.08***  (2.09, 4.58) | 9.45***  (7.36, 12.12) | 0.33***  (0.25, 0.46) | 2.19***  (1.85, 2.59) |  |
|  | Other self-reported mental health diagnosis | | 1.20  (0.96, 1.51) | 1.35***  (1.13, 1.62) | 0.86*  (0.76, 0.98) | 1.40***  (1.30, 1.51) | 1.39  (0.95, 2.03) | 1.26  (0.95, 1.68) | 1.06  (0.86, 1.31) | 1.28***  (1.14, 1.44) |  |
|  | Age of onset | | 0.72***  (0.67, 0.78) | 0.62***  (0.59, 0.66) | 1.26***  (1.19, 1.34) | 0.63***  (0.61, 0.65) | 0.84**  (0.74, 0.94) | 0.72***  (0.65, 0.79) | 1.34***  (1.21, 1.48) | 0.61***  (0.58, 0.64) |  |
|  | Recurrence | | 1.06***  (1.04, 1.09) | 1.03**  (1.01, 1.05) | 1.02**  (1.01, 1.04) | 1.08***  (1.07, 1.09) | 1.07**  (1.03, 1.11) | 1.03  (1.00, 1.06) | 1.03  (1.00, 1.06) | 1.08***  (1.06, 1.09) |  |
|  | Current depressive symptoms (PHQ9) | | 1.01  (0.99, 1.04) | 1.08***  (1.06, 1.10) | 0.93***  (0.92, 0.94) | 0.98***  (0.97, 0.99) | 0.99  (0.96, 1.03) | 1.07***  (1.04, 1.10) | 0.92***  (0.90, 0.94) | 0.98***  (0.97, 0.99) |  |
|  | Current anxiety symptoms (GAD7) | | 1.08***  (1.06, 1.11) | 1.00  (0.98, 1.02) | 1.08***  (1.07, 1.10) | 1.15***  (1.14, 1.16) | 1.10***  (1.06, 1.14) | 1.02  (0.99, 1.05) | 1.08***  (1.06, 1.11) | 1.15***  (1.14, 1.17) |  |
| Family history | | |  |  |  |  |  |  |  |  |  |
|  | Family history: Anxiety/depressive disorder | | 1.19  (0.95, 1.49) | 1.53***  (1.33, 1.77) | 0.77**  (0.65, 0.93) | 1.34***  (1.23, 1.45) | 1.09  (0.76, 1.56) | 1.12  (0.88, 1.43) | 0.94  (0.75, 1.18) | 0.96  (0.86, 1.08) |  |
|  | Family history: Other mental health disorder | | 1.25  (1.00, 1.57) | 1.47***  (1.26, 1.71) | 0.85  (0.71, 1.01) | 1.55***  (1.43, 1.69) | 0.86  (0.59, 1.24) | 1.00  (0.77, 1.31) | 0.96  (0.78, 1.19) | 1.09  (0.97, 1.21) |  |

STable 13. Results from partially adjusted and fully adjusted logistic regression models for comorbidity group analyses including family history. The table displays the odds ratios and confidence intervals for the logistic regression models for each comparison indicated in the column headers. The partially adjusted models refer to the regressions run with each group of independent variables separately (e.g., sociodemographic variables only). Results from the partially adjusted models, aside from the family history model, are the same as the main analysis. The fully adjusted models include all variables. The significance values (q) were adjusted using the false discovery rate multiple testing correction. Note that confidence intervals, while reported, are not fully informative due to the adjustment for multiple testing.

*Abbreviations:* Anx = Single anxiety; Anx-anx = Anxiety-anxiety; Anx-MDD = Anxiety-MDD; OR = odds ratio. CI = confidence interval. PHQ9 = 9-item patient health questionnaire. GAD7 = 7-item generalised anxiety disorder assessment.

*q < 0.05, **q < 0.01, ***q < 0.001

## STable 14. Results from partially adjusted and fully adjusted logistic regression models for temporal sequence comparisons, including self-reported family history.

|  | |  | Anxiety-first (vs MDD-first) | |
| --- | --- | --- | --- | --- |
| Variable | | | Partially adjusted model  OR (95% CI) | Fully adjusted model  OR (95% CI) |
| Sociodemographic | | |  |  |
|  | Age | | 0.98 (0.96, 1.00) | 1.25*** (1.20, 1.31) |
|  | Female | | 1.44*** (1.33, 1.56) | 0.99 (0.84, 1.16) |
|  | University degree | | 0.69*** (0.57, 0.84) | 1.72* (1.13, 2.61) |
|  | A-levels or equivalent | | 0.76* (0.62, 0.93) | 1.53 (1.00, 2.32) |
|  | GCSEs or equivalent | | 0.82 (0.67, 0.99) | 1.34 (0.89, 2.01) |
|  | White | | 1.15 (1.01, 1.31) | 1.09 (0.83, 1.43) |
| Trauma | | |  |  |
|  | Childhood trauma | | 1.10** (1.04, 1.18) | 0.86* (0.76, 0.97) |
|  | Adult trauma | | 1.01 (0.95, 1.08) | 0.85* (0.75, 0.96) |
|  | Catastrophic trauma | | 1.00 (0.93, 1.07) | 0.92 (0.81, 1.06) |
| Clinical | | |  |  |
|  | Self-reported anxiety/depressive disorder diagnosis | | 0.75** (0.62, 0.91) | 0.51*** (0.38, 0.69) |
|  | Other self-reported mental health diagnosis | | 0.79*** (0.74, 0.85) | 0.85* (0.75, 0.95) |
|  | Age of onset | | 0.15*** (0.14, 0.17) | 0.15*** (0.13, 0.17) |
|  | Recurrence | | 0.98*** (0.97, 0.99) | 0.96*** (0.95, 0.98) |
|  | Current depressive symptoms (PHQ9) | | 0.96*** (0.95, 0.97) | 0.97*** (0.96, 0.98) |
|  | Current anxiety symptoms (GAD7) | | 1.05*** (1.05, 1.06) | 1.06*** (1.04, 1.07) |
| Family history | | |  |  |
|  | Family history: Anxiety/depressive disorder | | 1.06 (0.96, 1.18) | 0.99 (0.87, 1.14) |
|  | Family history: Other mental health disorder | | 1.09 (0.99, 1.20) | 0.96 (0.85, 1.08) |

STable 14. Results from partially adjusted and fully adjusted logistic regression models for temporal sequence analysis including family history. The table displays the odds ratios and confidence intervals for the logistic regression models. The partially adjusted models refer to the regressions run with each group of independent variables separately (e.g., sociodemographic variables only). Results from the partially adjusted models, aside from the family history model, are the same as the main analysis. The fully adjusted models include all variables. The significance values (q) were adjusted using the false discovery rate multiple testing correction. Note that confidence intervals, while reported, are not fully informative due to the adjustment for multiple testing.

*Abbreviations:* OR = odds ratio. CI = confidence interval. PHQ9 = 9-item patient health questionnaire. GAD7 = 7-item generalised anxiety disorder assessment.

*q < 0.05, **q < 0.01, ***q < 0.001

## STable 15. Results from partially adjusted and fully adjusted logistic regression models for temporal sequence comparisons, including self-reported family history and excluding age of onset and current anxiety symptoms.

|  | |  | Anxiety-first (vs MDD-first) | |
| --- | --- | --- | --- | --- |
| Variable | | | Partially adjusted model  OR (95% CI) | Fully adjusted model  OR (95% CI) |
| Sociodemographic | | |  |  |
|  | Age | | 0.98 (0.96, 1.00) | 1.01 (0.97, 1.04) |
|  | Female | | 1.44*** (1.33, 1.56) | 1.30** (1.13, 1.49) |
|  | University degree | | 0.69*** (0.57, 0.84) | 1.31 (0.91, 1.89) |
|  | A-levels or equivalent | | 0.76* (0.62, 0.93) | 1.26 (0.88, 1.82) |
|  | GCSEs or equivalent | | 0.82 (0.67, 0.99) | 1.17 (0.82, 1.68) |
|  | White | | 1.15 (1.01, 1.31) | 1.15 (0.90, 1.47) |
| Trauma | | |  |  |
|  | Childhood trauma | | 1.10** (1.04, 1.18) | 1.10 (0.99, 1.23) |
|  | Adult trauma | | 1.01 (0.95, 1.08) | 0.93 (0.84, 1.04) |
|  | Catastrophic trauma | | 1.00 (0.93, 1.07) | 1.03 (0.91, 1.15) |
| Clinical | | |  |  |
|  | Self-reported anxiety/depressive disorder diagnosis | | 0.79* (0.67, 0.94) | 0.62** (0.48, 0.79) |
|  | Other self-reported mental health diagnosis | | 1.05 (0.98, 1.12) | 1.03 (0.93, 1.14) |
|  | Recurrence | | 1.03*** (1.02, 1.03) | 1.02* (1.01, 1.04) |
|  | Current depressive symptoms (PHQ9) | | 1.00 (1.00, 1.01) | 1.00 (0.99, 1.01) |
| Family history | | |  |  |
|  | Family history: Anxiety/depressive disorder | | 1.06 (0.96, 1.18) | 1.06 (0.94, 1.20) |
|  | Family history: Other mental health disorder | | 1.09 (0.99, 1.20) | 1.08 (0.97, 1.21) |

STable 15. Results from partially adjusted and fully adjusted logistic regression models for temporal sequence analysis including family history and excluding age of onset. The table displays the odds ratios and confidence intervals for the logistic regression models. The partially adjusted models refer to the regressions run with each group of independent variables separately (e.g., sociodemographic variables only). The clinical model is the only partially adjusted analysis with different results from the model in STable 12, as it now excludes age of onset and GAD7. The fully adjusted models include all variables. The significance values (q) were adjusted using the false discovery rate multiple testing correction. Note that confidence intervals, while reported, are not fully informative due to the adjustment for multiple testing.

*Abbreviations:* OR = odds ratio. CI = confidence interval. PHQ9 = 9-item patient health questionnaire. GAD7 = 7-item generalised anxiety disorder assessment.

*q < 0.05, **q < 0.01, ***q < 0.001

## STable 16. Results from partially adjusted and fully adjusted logistic regression models for comorbidity group comparisons, including genetic factors.

|  | |  | Partially adjusted models | | | | Fully adjusted models | | | |
| --- | --- | --- | --- | --- | --- | --- | --- | --- | --- | --- |
| Variable | | | Anx-anx  (vs Anx)  OR (95% CI) | Anx-MDD  (vs Anx)  OR (95% CI) | Anx-anx  (vs Anx-MDD)  OR (95% CI) | Anx-MDD  (vs MDD only)  OR (95% CI) | Anx-anx  (vs Anx)  OR (95% CI) | Anx-MDD  (vs Anx)  OR (95% CI) | Anx-anx  (vs Anx-MDD)  OR (95% CI) | Anx-MDD  (vs MDD only)  OR (95% CI) |
| Sociodemographic | | |  |  |  |  |  |  |  |  |
|  | Age | | 0.77***  (0.73, 0.81) | 0.84***  (0.81, 0.87) | 0.89***  (0.85, 0.92) | 0.80***  (0.78, 0.82) | 0.97  (0.82, 1.14) | 1.13  (1.01, 1.27) | 0.77***  (0.70, 0.85) | 1.07*  (1.02, 1.12) |
|  | Female | | 1.14  (0.95, 1.35) | 1.45***  (1.28, 1.64) | 0.74***  (0.66, 0.84) | 1.37***  (1.28, 1.47) | 1.74  (1.12, 2.73) | 1.32  (0.97, 1.78) | 0.92  (0.71, 1.21) | 1.08  (0.95, 1.23) |
|  | University degree | | 0.51**  (0.32, 0.81) | 0.71  (0.49, 0.99) | 0.84  (0.63, 1.15) | 0.39***  (0.31, 0.49) | 1.20  (0.34, 4.23) | 1.72  (0.67, 3.98) | 0.86  (0.45, 1.83) | 0.76  (0.44, 1.27) |
|  | A-levels or equivalent | | 0.61  (0.37, 0.98) | 0.87  (0.59, 1.23) | 0.77  (0.57, 1.07) | 0.53***  (0.42, 0.66) | 0.65  (0.18, 2.32) | 1.39  (0.54, 3.20) | 0.57  (0.29, 1.22) | 0.77  (0.45, 1.29) |
|  | GCSEs or equivalent | | 0.90  (0.56, 1.46) | 0.95  (0.65, 1.34) | 1.08  (0.80, 1.50) | 0.64***  (0.50, 0.80) | 0.90  (0.27, 3.04) | 1.47  (0.59, 3.29) | 0.63  (0.33, 1.33) | 0.56  (0.33, 0.92) |
|  | White | | 1.09  (0.78, 1.53) | 1.03  (0.79, 1.30) | 1.07  (0.86, 1.37) | 1.17**  ( 1.03, 1.33) | 0.73 (0.04, 8.25) | 0.86  (0.11, 3.64) | 1.12  (0.38, 4.81) | 1.05  (0.58, 1.83) |
| Trauma | | |  |  |  |  |  |  |  |  |
|  | Childhood trauma | | 1.39***  (1.19, 1.62) | 1.78***  (1.59, 2.00) | 0.78***  (0.70, 0.87) | 1.79***  (1.69, 1.90) | 0.93  (0.61, 1.42) | 1.08  (0.81, 1.45) | 0.87  (0.69, 1.10) | 1.13  (1.01, 1.27) |
|  | Adult trauma | | 1.63***  (1.38, 1.92) | 2.16***  (1.91, 2.45) | 0.75***  (0.67, 0.84) | 1.67***  (1.57, 1.77) | 1.11  (0.70, 1.77) | 1.39  (1.01, 1.92) | 0.79  (0.62, 1.00) | 1.23**  (1.09, 1.39) |
|  | Catastrophic trauma | | 1.01  (0.87, 1.19) | 1.45***  (1.30, 1.63) | 0.69***  (0.62, 0.78) | 1.11***  (1.04, 1.18) | 0.83  (0.54, 1.26) | 1.50*  (1.13, 2.00) | 0.65**  (0.51, 0.82) | 1.08  (0.96, 1.22) |
| Clinical | | |  |  |  |  |  |  |  |  |
|  | Self-reported anxiety/depressive disorder diagnosis | | 3.10***  (2.35, 4.10) | 7.98***  (6.63, 9.58) | 0.39***  (0.32, 0.49) | 2.20***  (1.94, 2.48) | 1.43  (0.77, 2.63) | 5.68***  (4.02, 8.02) | 0.30***  (0.21, 0.45) | 1.92***  (1.55, 2.38) |
|  | Other self-reported mental health diagnosis | | 1.20  (0.96, 1.51) | 1.35***  (1.13, 1.62) | 0.86*  (0.76, 0.98) | 1.40***  (1.30, 1.51) | 1.41  (0.88, 2.25) | 1.39  (0.99, 1.97) | 0.91  (0.72, 1.15) | 1.29***  (1.14, 1.46) |
|  | Age of onset | | 0.72***  (0.67, 0.78) | 0.62***  (0.59, 0.66) | 1.26***  (1.19, 1.34) | 0.63***  (0.61, 0.65) | 0.64***  (0.54, 0.75) | 0.59***  (0.53, 0.66) | 1.40***  (1.24, 1.57) | 0.62***  (0.59, 0.66) |
|  | Recurrence | | 1.06***  (1.04, 1.09) | 1.03**  (1.01, 1.05) | 1.02**  (1.01, 1.04) | 1.08***  (1.07, 1.09) | 1.09**  (1.04, 1.14) | 1.02  (0.99, 1.05) | 1.04  (1.01, 1.07) | 1.06***  (1.04, 1.07) |
|  | Current depressive symptoms (PHQ9) | | 1.01  (0.99, 1.04) | 1.08***  (1.06, 1.10) | 0.93***  (0.92, 0.94) | 0.98***  (0.97, 0.99) | 0.99 (0.94, 1.03) | 1.04  (1.00, 1.07) | 0.93***  (0.90, 0.95) | 0.98***  (0.96, 0.99) |
|  | Current anxiety symptoms (GAD7) | | 1.08***  (1.06, 1.11) | 1.00  (0.98, 1.02) | 1.08***  (1.07, 1.10) | 1.15***  (1.14, 1.16) | 1.07*  (1.02, 1.12) | 1.01  (0.98, 1.05) | 1.08***  (1.05, 1.10) | 1.15***  (1.13, 1.17) |
| Genetic | | |  |  |  |  |  |  |  |  |
|  | Anxiety PRS | | 1.00  (0.88, 1.14) | 0.96  (0.88, 1.05) | 1.04  (0.94, 1.14) | 1.03  (0.98, 1.08) | 0.95  (0.77, 1.18) | 0.94  (0.81, 1.09) | 1.01  (0.90, 1.15) | 1.05  (0.99, 1.11) |
|  | MDD PRS | | 1.03  (0.92, 1.16) | 0.98  (0.90, 1.06) | 1.02  (0.94, 1.11) | 1.00  (0.96, 1.04) | 0.93  (0.76, 1.13) | 0.89  (0.77, 1.02) | 1.04  (0.93, 1.16) | 1.01  (0.96, 1.07) |
|  | Neuroticism PRS | | 1.08  (0.94, 1.23) | 1.09  (1.00, 1.20) | 0.98  (0.90, 1.08) | 1.05  (1.00, 1.10) | 1.40*  (1.10, 1.78) | 1.19  (1.02, 1.38) | 1.04  (0.93, 1.18) | 1.03  (0.97, 1.09) |
|  | ADHD PRS | | 1.03  (0.91, 1.17) | 1.04  (0.96, 1.13) | 1.02  (0.93, 1.11) | 1.03  (0.99, 1.08) | 1.06  (0.87, 1.30) | 1.03  (0.90, 1.18) | 1.09  (0.97, 1.22) | 1.00  (0.94, 1.06) |
|  | Autism PRS | | 1.09  (0.96, 1.24) | 1.01  (0.92, 1.10) | 1.07  (0.98, 1.17) | 1.00  (0.96, 1.04) | 1.03  (0.83, 1.28) | 0.92  (0.80, 1.06) | 1.10  (0.98, 1.24) | 0.95  (0.90, 1.01) |
|  | Schizophrenia PRS | | 0.96  (0.84, 1.09) | 0.98  (0.90, 1.07) | 0.96  (0.87, 1.05) | 1.00  (0.95, 1.05) | 0.93  (0.74, 1.15) | 0.92  (0.79, 1.06) | 0.99  (0.88, 1.11) | 0.99  (0.93, 1.05) |
|  | Educational attainment PRS | | 0.87  (0.77, 0.99) | 0.88*  (0.81, 0.96) | 0.98  (0.90, 1.07) | 0.93**  (0.89, 0.97) | 1.00  (0.81, 1.23) | 0.96  (0.84, 1.11) | 0.96  (0.86, 1.08) | 1.01  (0.95, 1.07) |
| Covariates | | |  |  |  |  |  |  |  |  |
|  | GLAD genotyping batch set 1 | | 0.93  (0.62, 1.38) | 1.04  (0.77, 1.39) | 0.88  (0.67, 1.15) | 0.91  (0.79, 1.04) | 0.83  (0.33, 2.08) | 0.93  (0.45, 1.88) | 0.95  (0.63, 1.41) | 0.85  (0.71, 1.02) |
|  | GLAD genotyping batch set 2 | | 0.93  (0.65, 1.34) | 0.72  (0.55, 0.95) | 1.30  (1.02, 1.65) | 0.75***  (0.65, 0.85) | 0.45  (0.20, 0.96) | 0.33**  (0.18, 0.59) | 1.83**  (1.31, 2.61) | 0.61***  (0.51, 0.73) |
|  | GLAD genotyping batch set 3 | | 1.09  (0.75, 1.59) | 0.85  (0.64, 1.13) | 1.26  (0.99, 1.61) | 0.78***  (0.68, 0.89) | 0.39  (0.18, 0.83) | 0.32***  (0.17, 0.55) | 1.86**  (1.33, 2.64) | 0.60***  (0.50, 0.71) |
|  | COPING NBR batch set 1 | | 0.19***  (0.11, 0.33) | 0.12***  (0.08, 0.16) | 1.78  (1.11, 2.77) | 0.16***  (0.13, 0.19) | 0.07***  (0.02, 0.21) | 0.08***  (0.04, 0.16) | 1.69  (0.77, 3.44) | 0.50***  (0.36, 0.69) |
|  | COPING NBR batch set 2 | | 0.17***  (0.08, 0.32) | 0.12***  (0.08, 0.17) | 1.47  (0.79, 2.57) | 0.22***  (0.17, 0.27) | 0.18**  (0.06, 0.50) | 0.16***  (0.08, 0.31) | 2.02  (1.10, 3.57) | 0.42***  (0.32, 0.55) |
|  | COPING NBR batch set 3 | | 0.28***  (0.16, 0.47) | 0.13***  (0.10, 0.19) | 2.01*  (1.28, 3.06) | 0.19***  (0.16, 0.23) | 0.09***  (0.03, 0.25) | 0.10***  (0.05, 0.19) | 2.18*  (1.18, 3.88) | 0.39***  (0.30, 0.50) |
|  | Principal component 1 | | 0.01  (0.00, 1130.67) | 46.25  (0.03, 200659.25) | 0.00  (0.00, 9.52) | 0.16  (0.00, 6.37) | 0.00  (0.00, 902313.78) | 5.55  (0.00, 1767838.84) | 0.00  (0.00, 109.13) | 0.08  (0.00, 9.47) |
|  | Principal component 2 | | 192263456.90 (3.88, 11503282536106844.00) | 263342684.07** (1215.26, 78391457200923.31) | 0.95  (0.00, 111096.17) | 15.54  (0.04, 6238.47) | 9641388915331758.00 (158.05, 1853913552884084535554094399488.00) | 282176197812196.31* (242180.22, 830811130846418846089216.00) | 0.00 (0.00, 2487.53) | 9.63 (0.00, 21279.76) |
|  | Principal component 3 | | 0.05  (0.00, 26855142.67) | 261.38 (0.00, 164731230.67) | 0.04 (0.00, 14993.35) | 0.10 (0.00, 56.24) | 0.01 (0.00, 576853810113029.75) | 711.21 (0.00, 1937083044713.03) | 0.00 (0.00, 51.89) | 0.03 (0.00, 133.75) |
|  | Principal component 4 | | 36827.97 (0.00, 406503765260906135552.00) | 0.03 (0.00, 480373.76) | 4687907.81 (0.00, 192944208995156852736.00) | 44.74 (0.00, 1573847.29) | 0.42 (0.00, 30622226031821217792.00) | 0.00 (0.00, 102927691.04) | 28.81 (0.00, 100114340410235680.00) | 0.03 (0.00, 11567.79) |
|  | Principal component 5 | | 18.60 (0.00, 24355224885386.24) | 4.06 (0.00, 23069339.42) | 1.16 (0.00, 5189253988.19) | 0.01 (0.00, 28.66) | 0.00 (0.00, 146457806.86) | 0.00 (0.00, 13119.60) | 3.95 (0.00, 2804231107023.78) | 0.00 (0.00, 58.81) |
|  | Principal component 6 | | 61.63 (0.00, 82219999946.17) | 5425.66 (0.00, 55961645860.50) | 0.23 (0.00, 711036.18) | 0.00 (0.00, 1.34) | 259.89 (0.00, 13305004804583360.00) | 7.84 (0.00, 1044537926812.28) | 66.11 (0.00, 7030985818.50) | 0.02 (0.00, 199.16) |
|  | Principal component 7 | | 0.00 (0.00, 150512.35) | 2.25 (0.00, 730286791.35) | 0.00 (0.00, 515.91) | 23.01 (0.00, 1558778.16) | 0.00 (0.00, 11373854.39) | 0.01 (0.00, 14112008681.28) | 0.00 (0.00, 14646.55) | 0.01 (0.00, 10383.98) |
|  | Principal component 8 | | 0.00 (0.00, 1111312494.22) | 0.00 (0.00, 0.10) | 770.90 (0.00, 306760576222.93) | 12.29 (0.00, 194064.52) | 0.00 (0.00, 146551489199.07) | 0.00 (0.00, 0.02) | 361352.44 (0.00, 68915639205150272.00) | 1188.44 (0.01, 225591064.22) |
|  | Principal component 9 | | 0.04 (0.00, 14389679153705.53) | 1442.53 (0.00, 330253477536.65) | 0.05 (0.00, 1902524661.61) | 34.63 (0.00, 2535993.55) | 0.00 (0.00, 25732069001338.92) | 0.60 (0.00, 1904061692959.27) | 9.49 (0.00, 217700345663864.66) | 0.13 (0.00, 203060.86) |
|  | Principal component 10 | | 0.00 (0.00, 51961928.80) | 0.06 (0.00, 3723492.50) | 0.03 (0.00, 2671419.77) | 0.17 (0.00, 1784.82) | 0.01 (0.00, 40381995401634296.00) | 0.08 (0.00, 1157402408107.74) | 0.00 (0.00, 32692082.21) | 0.24 (0.00, 38060.40) |

STable 16. Results from partially adjusted and fully adjusted logistic regression models for comorbidity group analyses including genetic factors. The table displays the odds ratios and confidence intervals for the logistic regression models for each comparison indicated in the column headers. The partially adjusted models refer to the regressions run with each group of independent variables separately (e.g., sociodemographic variables only). Results from the partially adjusted models, aside from the genetic factors model, are the same as the main analysis. The partially adjusted genetic model included both the polygenic scores and the covariates. The fully adjusted models include all variables. The significance values (q) were adjusted using the false discovery rate multiple testing correction. Note that confidence intervals, while reported, are not fully informative due to the adjustment for multiple testing.

*Abbreviations:* Anx = Single anxiety. Anx-anx = Anxiety-anxiety. Anx-MDD = Anxiety-MDD. OR = odds ratio. CI = confidence interval. PHQ9 = 9-item patient health questionnaire. GAD7 = 7-item generalised anxiety disorder assessment. PRS = polygenic risk scores. MDD = major depressive disorder. ADHD = attention deficit and hyperactivity disorder.

*q < 0.05, **q < 0.01, ***q < 0.001

## STable 17. Results from partially adjusted and fully adjusted logistic regression models for temporal sequence comparisons, including genetic factors.

|  | | Anxiety-first (vs MDD-first) | |
| --- | --- | --- | --- |
| Variable | | Partially adjusted model  OR (95% CI) | Fully adjusted model  OR (95% CI) |
| Sociodemographic | |  |  |
|  | Age | 0.98 (0.96, 1.00) | 1.22*** (1.16, 1.28) |
|  | Female | 1.44*** (1.33, 1.56) | 1.20 (1.02, 1.42) |
|  | University degree | 0.69*** (0.57, 0.84) | 1.47 (0.89, 2.42) |
|  | A-levels or equivalent | 0.76* (0.62, 0.93) | 1.19 (0.72, 1.95) |
|  | GCSEs or equivalent | 0.82 (0.67, 0.99) | 1.10 (0.67, 1.79) |
|  | White | 1.15 (1.01, 1.31) | 1.01 (0.56, 1.85) |
| Trauma | |  |  |
|  | Childhood trauma | 1.10** (1.03, 1.18) | 0.96 (0.83, 1.10) |
|  | Adult trauma | 1.01 (0.95, 1.08) | 0.88 (0.76, 1.00) |
|  | Catastrophic trauma | 1.00 (0.93, 1.07) | 0.79** (0.68, 0.92) |
| Clinical | |  |  |
|  | Self-reported anxiety/depressive disorder diagnosis | 0.75** (0.62, 0.91) | 1.05 (0.71, 1.53) |
|  | Other self-reported mental health diagnosis | 0.79*** (0.74, 0.85) | 0.90 (0.79, 1.02) |
|  | Age of onset | 0.15*** (0.14, 0.17) | 0.10*** (0.08, 0.11) |
|  | Recurrence | 0.98*** (0.97, 0.99) | 0.94*** (0.92, 0.95) |
|  | Current depressive symptoms (PHQ9) | 0.96*** (0.95, 0.97) | 0.98*** (0.96, 0.99) |
|  | Current anxiety symptoms (GAD7) | 1.05*** (1.05, 1.06) | 1.05*** (1.03, 1.06) |
| Genetic | |  |  |
|  | Anxiety PRS | 0.98 (0.93, 1.03) | 0.99 (0.92, 1.06) |
|  | MDD PRS | 1.03 (0.98, 1.08) | 1.04 (0.97, 1.10) |
|  | Neuroticism PRS | 1.00 (0.95, 1.05) | 1.00 (0.93, 1.07) |
|  | ADHD PRS | 1.01 (0.96, 1.06) | 1.03 (0.96, 1.10) |
|  | Autism PRS | 0.99 (0.94, 1.04) | 0.95 (0.89, 1.01) |
|  | Schizophrenia PRS | 0.97 (0.92, 1.02) | 0.95 (0.88, 1.01) |
|  | Educational attainment PRS | 0.97 (0.93, 1.02) | 0.96 (0.90, 1.03) |
| Covariates | |  |  |
|  | GLAD genotyping batch set 1 | 1.00 (0.87, 1.14) | 1.04 (0.86, 1.26) |
|  | GLAD genotyping batch set 2 | 1.38*** (1.21, 1.58) | 1.48*** (1.23, 1.78) |
|  | GLAD genotyping batch set 3 | 1.27** (1.11, 1.45) | 1.74*** (1.45, 2.10) |
|  | COPING NBR batch set 1 | 2.73*** (2.02, 3.72) | 6.25*** (3.75, 10.64) |
|  | COPING NBR batch set 2 | 3.01*** (2.08, 4.41) | 10.27*** (6.27, 17.22) |
|  | COPING NBR batch set 3 | 2.50*** (1.84, 3.43) | 6.30*** (4.10, 9.79) |
|  | Principal component 1 | 0.37 (0.00, 30.79) | 1.60 (0.00, 1139.65) |
|  | Principal component 2 | 0.00 (0.00, 1.15) | 0.07 (0.00, 670.84) |
|  | Principal component 3 | 0.00* (0.00, 0.12) | 0.00 (0.00, 85.25) |
|  | Principal component 4 | 28.10 (0.00, 364635728.34) | 0.00 (0.00, 17411743.77) |
|  | Principal component 5 | 0.29 (0.00, 39410.67) | 0.05 (0.00, 699909.27) |
|  | Principal component 6 | 0.51 (0.00, 2806.02) | 0.13 (0.00, 11411.07) |
|  | Principal component 7 | 0.00 (0.00, 1611.83) | 81.23 (0.00, 18229570813.52) |
|  | Principal component 8 | 0.36 (0.00, 27077.63) | 0.01 (0.00, 27403.37) |
|  | Principal component 9 | 7744.24 (0.01, 10976189969.04) | 3954.17 (0.00, 1158362555252.31) |
|  | Principal component 10 | 0.53 (0.00, 15448.57) | 444.52 (0.00, 631379852.71) |

STable 17. Results from partially adjusted and fully adjusted logistic regression models for temporal sequence analysis including genetic factors. The table displays the odds ratios and confidence intervals for the logistic regression models. The partially adjusted models refer to the regressions run with each group of independent variables separately (e.g., sociodemographic variables only). Results from the partially adjusted models, aside from the genetic factors model, are the same as the analysis presented in the manuscript (Table 2). The fully adjusted models include all variables. The significance values (q) were adjusted using the false discovery rate multiple testing correction. Note that confidence intervals, while reported, are not fully informative due to the adjustment for multiple testing.

*Abbreviations:* OR = odds ratio. CI = confidence interval. PHQ9 = 9-item patient health questionnaire. GAD7 = 7-item generalised anxiety disorder assessment. PRS = polygenic risk scores. MDD = major depressive disorder. ADHD = attention deficit and hyperactivity disorder.

*q < 0.05, **q < 0.01, ***q < 0.001

## STable 18. Results from partially adjusted and fully adjusted logistic regression models for temporal sequence comparisons, including genetic factors and excluding age of onset and current anxiety symptoms.

|  | | |  | Anxiety-first (vs MDD-first) | |
| --- | --- | --- | --- | --- | --- |
| Variable | | Partially adjusted model  OR (95% CI) | | | Fully adjusted model  OR (95% CI) |
| Sociodemographic | |  | | |  |
|  | Age | 0.98 (0.96, 1.00) | | | 0.97 (0.93, 1.01) |
|  | Female | 1.44*** (1.33, 1.56) | | | 1.55*** (1.35, 1.78) |
|  | University degree | 0.69*** (0.57, 0.84) | | | 1.22 (0.80, 1.85) |
|  | A-levels or equivalent | 0.76* (0.62, 0.93) | | | 1.06 (0.70, 1.61) |
|  | GCSEs or equivalent | 0.82 (0.67, 0.99) | | | 1.05 (0.70, 1.59) |
|  | White | 1.15 (1.01, 1.31) | | | 0.89 (0.53, 1.50) |
| Trauma | |  | | |  |
|  | Childhood trauma | 1.10** (1.03, 1.18) | | | 1.22** (1.09, 1.37) |
|  | Adult trauma | 1.01 (0.95, 1.08) | | | 0.94 (0.84, 1.06) |
|  | Catastrophic trauma | 1.00 (0.93, 1.07) | | | 0.93 (0.82, 1.05) |
| Clinical | |  | | |  |
|  | Self-reported anxiety/depressive disorder diagnosis | 0.79* (0.67, 0.94) | | | 0.96 (0.70, 1.31) |
|  | Other self-reported mental health diagnosis | 1.05 (0.98, 1.12) | | | 1.14 (1.02, 1.27) |
|  | Recurrence | 1.03*** (1.02, 1.03) | | | 1.00 (0.99, 1.02) |
|  | Current depressive symptoms (PHQ9) | 1.00 (1.00, 1.01) | | | 1.00 (0.99, 1.01) |
| Genetic | |  | | |  |
|  | Anxiety PRS | 0.98 (0.93, 1.03) | | | 0.99 (0.93, 1.05) |
|  | MDD PRS | 1.03 (0.98, 1.08) | | | 1.03 (0.98, 1.09) |
|  | Neuroticism PRS | 1.00 (0.95, 1.05) | | | 0.99 (0.93, 1.05) |
|  | ADHD PRS | 1.01 (0.96, 1.06) | | | 1.01 (0.95, 1.06) |
|  | Autism PRS | 0.99 (0.94, 1.04) | | | 0.98 (0.93, 1.04) |
|  | Schizophrenia PRS | 0.97 (0.92, 1.02) | | | 0.98 (0.92, 1.03) |
|  | Educational attainment PRS | 0.97 (0.93, 1.02) | | | 0.97 (0.92, 1.03) |
| Covariates | |  | | |  |
|  | GLAD genotyping batch set 1 | 1.00 (0.87, 1.14) | | | 1.00 (0.85, 1.17) |
|  | GLAD genotyping batch set 2 | 1.38*** (1.21, 1.58) | | | 1.34** (1.14, 1.57) |
|  | GLAD genotyping batch set 3 | 1.27** (1.11, 1.45) | | | 1.49*** (1.27, 1.75) |
|  | COPING NBR batch set 1 | 2.68*** (1.99, 3.65) | | | 3.62*** (2.40, 5.55) |
|  | COPING NBR batch set 2 | 2.95*** (2.04, 4.33) | | | 3.80*** (2.61, 5.61) |
|  | COPING NBR batch set 3 | 2.48*** (1.83, 3.40) | | | 3.41*** (2.43, 4.82) |
|  | Principal component 1 | 0.37 (0.00, 30.73) | | | 0.66 (0.00, 141.77) |
|  | Principal component 2 | 0.00 (0.00, 1.21) | | | 0.00 (0.00, 4.02) |
|  | Principal component 3 | 0.00* (0.00, 0.11) | | | 0.00 (0.00, 1.41) |
|  | Principal component 4 | 21.73 (0.00, 284792736.40) | | | 0.20 (0.00, 14288612.82) |
|  | Principal component 5 | 0.22 (0.00, 29047.08) | | | 1.59 (0.00, 969550.04) |
|  | Principal component 6 | 0.44 (0.00, 2393.43) | | | 0.20 (0.00, 2840.13) |
|  | Principal component 7 | 0.00 (0.00, 2017.84) | | | 11.21 (0.00, 100204140.82) |
|  | Principal component 8 | 0.43 (0.00, 32668.17) | | | 0.15 (0.00, 51860.06) |
|  | Principal component 9 | 5323.46 (0.00, 7583973188.44) | | | 115.47 (0.00, 1066438605.98) |
|  | Principal component 10 | 0.64 (0.00, 18925.50) | | | 1.41 (0.00, 196950.43) |

STable 18. Results from partially adjusted and fully adjusted logistic regression models for temporal sequence analysis including genetic factors and excluding age of onset. The table displays the odds ratios and confidence intervals for the logistic regression models. The partially adjusted models refer to the regressions run with each group of independent variables separately (e.g., sociodemographic variables only). The clinical model is the only partially adjusted analysis with different results from the model in STable 15, as it now excludes age of onset. The fully adjusted models include all variables. The significance values (q) were adjusted using the false discovery rate multiple testing correction. Note that confidence intervals, while reported, are not fully informative due to the adjustment for multiple testing.

*Abbreviations:* OR = odds ratio. CI = confidence interval. PHQ9 = 9-item patient health questionnaire. GAD7 = 7-item generalised anxiety disorder assessment. PRS = polygenic risk scores. MDD = major depressive disorder. ADHD = attention deficit and hyperactivity disorder.

*q < 0.05, **q < 0.01, ***q < 0.001

# Supplementary Figures

## SFigure 1. Flowchart of genotyping quality control, imputation, and data exclusions


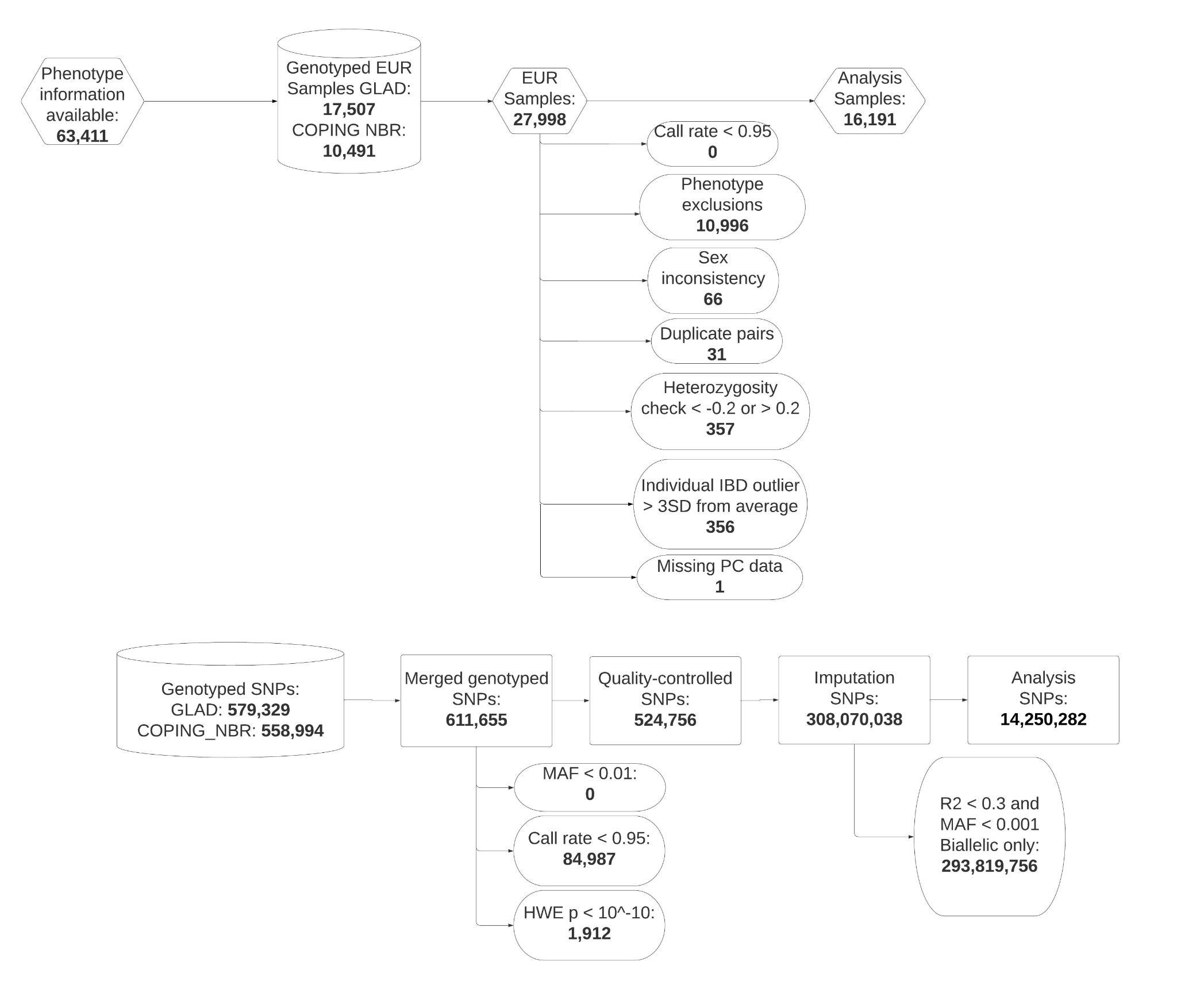


SFigure 1 displays the stages of genotyping quality control for individuals and genetic variants (SNPs) and the number of participants or variants excluded at each phase. The number of analysis samples indicated on the top right are individuals who met study eligibility criteria and had genetic data that passed all stages of quality control.

*Abbreviations:* EUR = European. GLAD = Genetic Links to Anxiety and Depression. COPING = COVID-19 Psychiatry and Neurological Genetics. NBR = NIHR BioResource. IBD = identity by descent. SD = standard deviation. PC = principal components. SNP = single nucleotide polymorphism. MAF = minor allele frequency. HWE = Hardy-Weinberg equilibrium.

## SFigure 2. Correlation matrix of all independent variables.


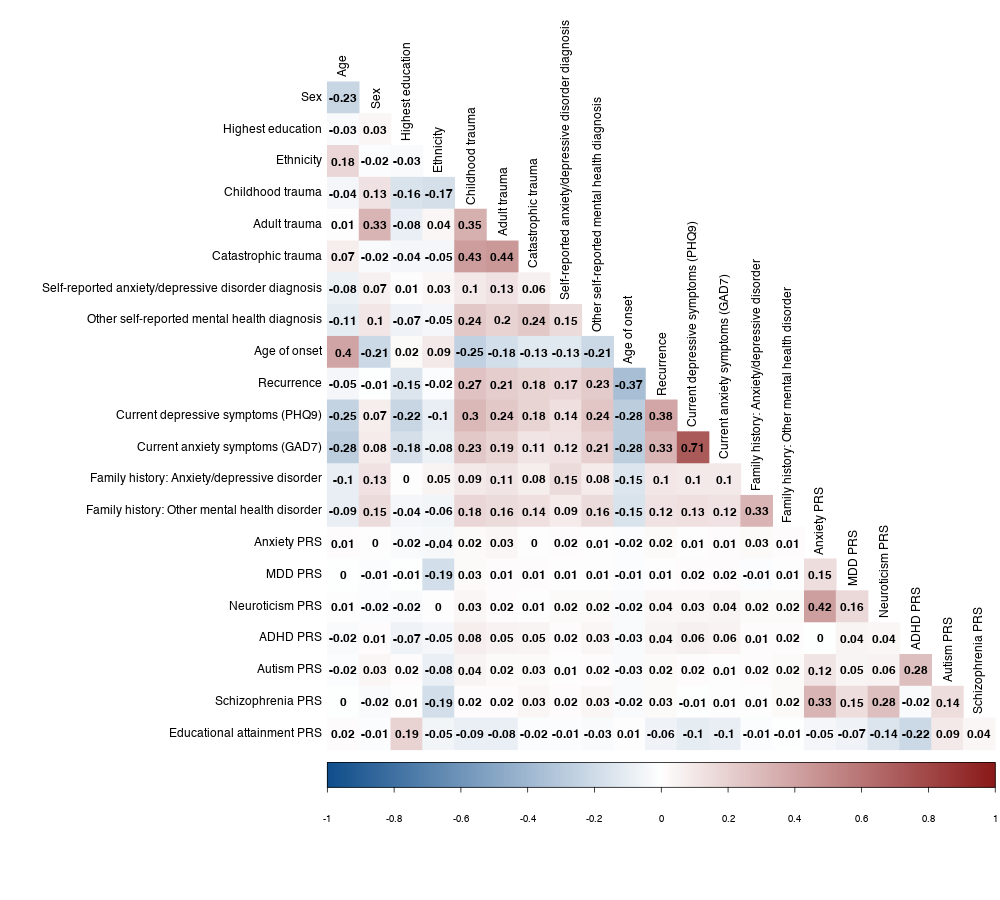


SFigure 2 displays a correlation matrix for all correlations between independent variables. Using the polycor package in R (https://cran.r-project.org/web/packages/polycor/polycor.pdf), correlations were calculated using Pearson’s correlation for numeric variables, polyserial correlations for a numeric and an ordinal variable, and polychoric correlations for ordinal variables. Positive correlations are displayed in red, while negative correlations are displayed in blue.

*Abbreviations:* PHQ9 = 9-item patient health questionnaire. GAD7 = 7-item generalised anxiety disorder assessment. PRS = polygenic risk score. MDD = major depressive disorder. ADHD = attention deficit and hyperactivity disorder.

## SFigure 3. Forest plot of results from the full logistic regression models for the comorbidity group comparisons.


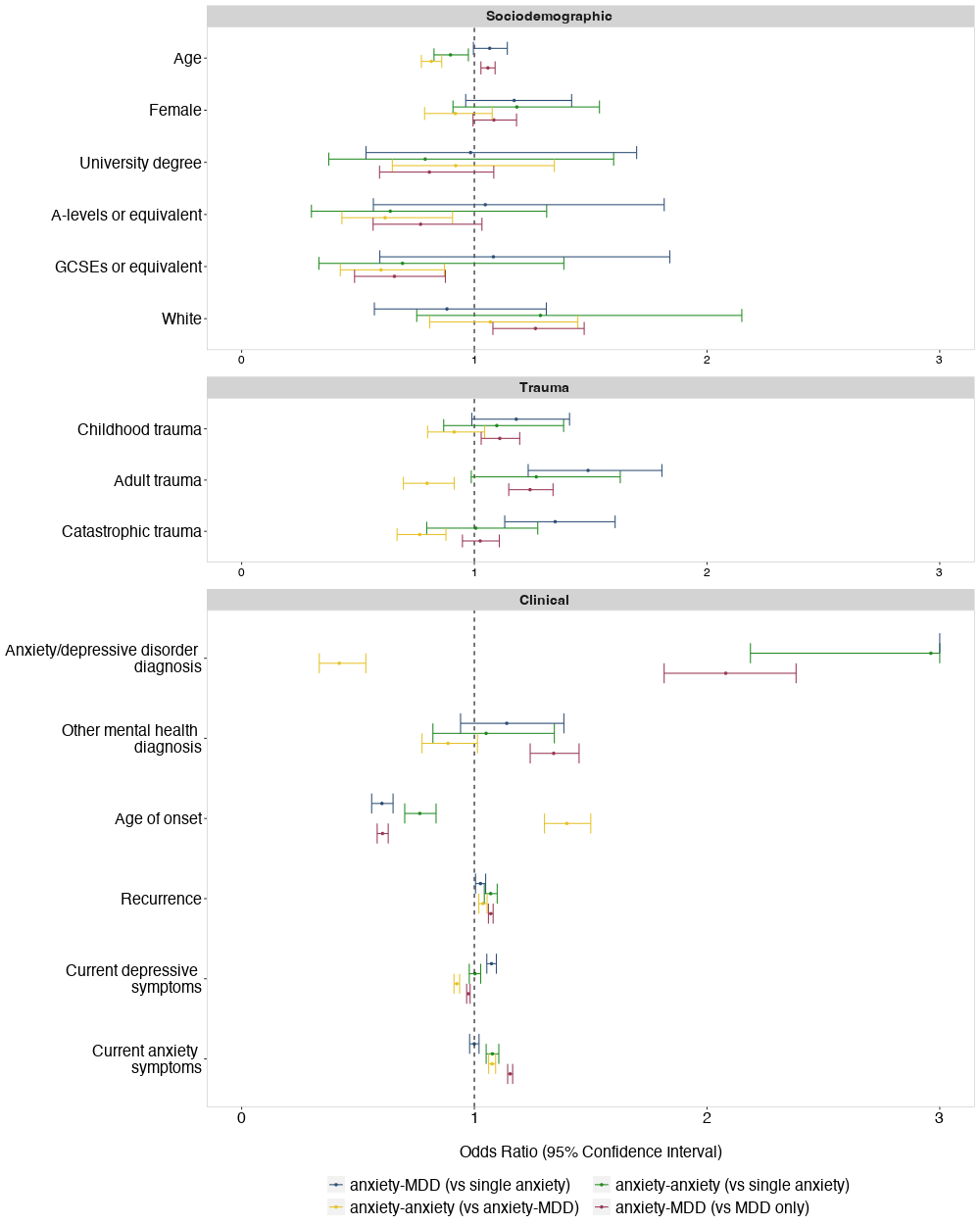


SFigure 3 displays the results from the fully adjusted models for each comorbidity group comparison. The range of the X-axis has been limited to three for viewing purposes. The odds ratio and confidence interval for anxiety/depressive disorder diagnosis for the anxiety-MDD vs single anxiety comparison (OR = 7.70; CI: 6.32, 9.35) has therefore been hidden, and the confidence interval for the anxiety-anxiety vs single anxiety comparison has been cut off (CI: 1.97, 3.60). For the categorical variables, the reference categories are as follows: Female (ref: Male); University, A-levels, and GCSEs (ref: No qualification); White (ref: Minoritised ethnic group); Childhood trauma (ref: No childhood trauma); Adult trauma (ref: No adult trauma); Catastrophic trauma (ref: No catastrophic trauma).

*Abbreviations:* MDD = major depressive disorder. OR = odds ratio. CI = confidence interval.

## SFigure 4. Forest plot of results from the full logistic regression model for the temporal sequence comparison.


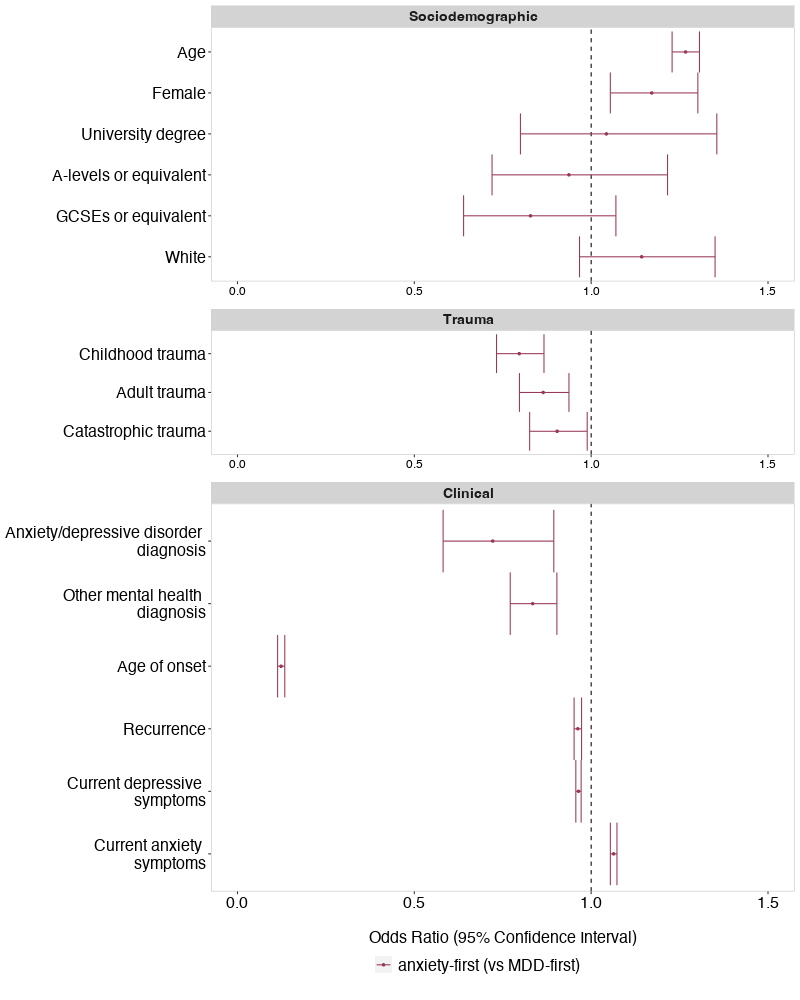


SFigure 4 displays the results from the fully adjusted models for the temporal sequence comparison.

Abbreviations: MDD = major depressive disorder.

## SFigure 5. Descriptives for self-reported family history by comorbidity group.


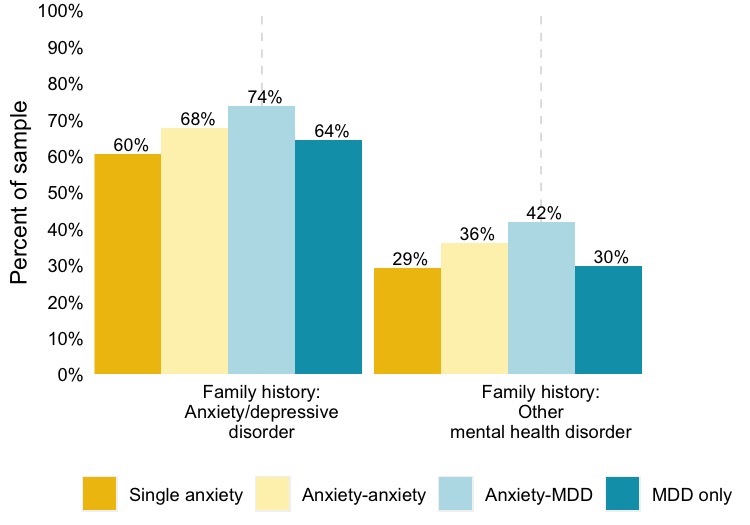


SFigure 5 displays the descriptives for self-reported family history by comorbidity group.

*Abbreviations:* MDD = major depressive disorder.

## SFigure 6. Descriptives for self-reported family history by temporal sequence.


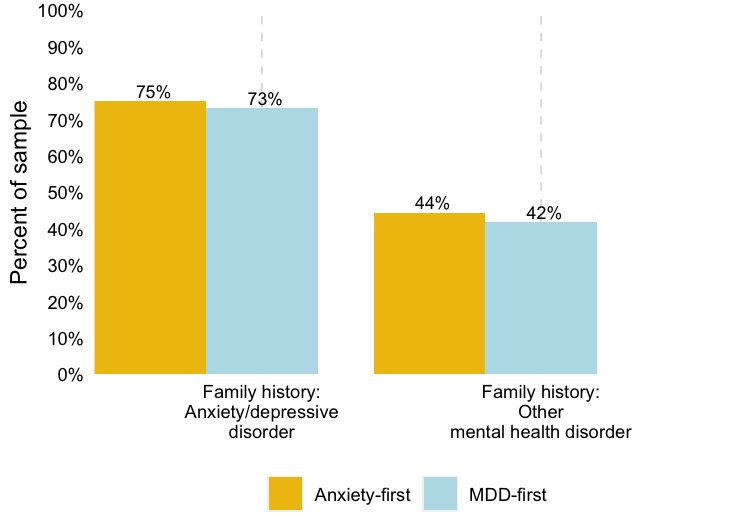


SFigure 6 displays the descriptives for self-reported family history by temporal sequence.

Abbreviations: MDD = major depressive disorder.

## SFigure 7. Descriptives for genetic factors by comorbidity group.


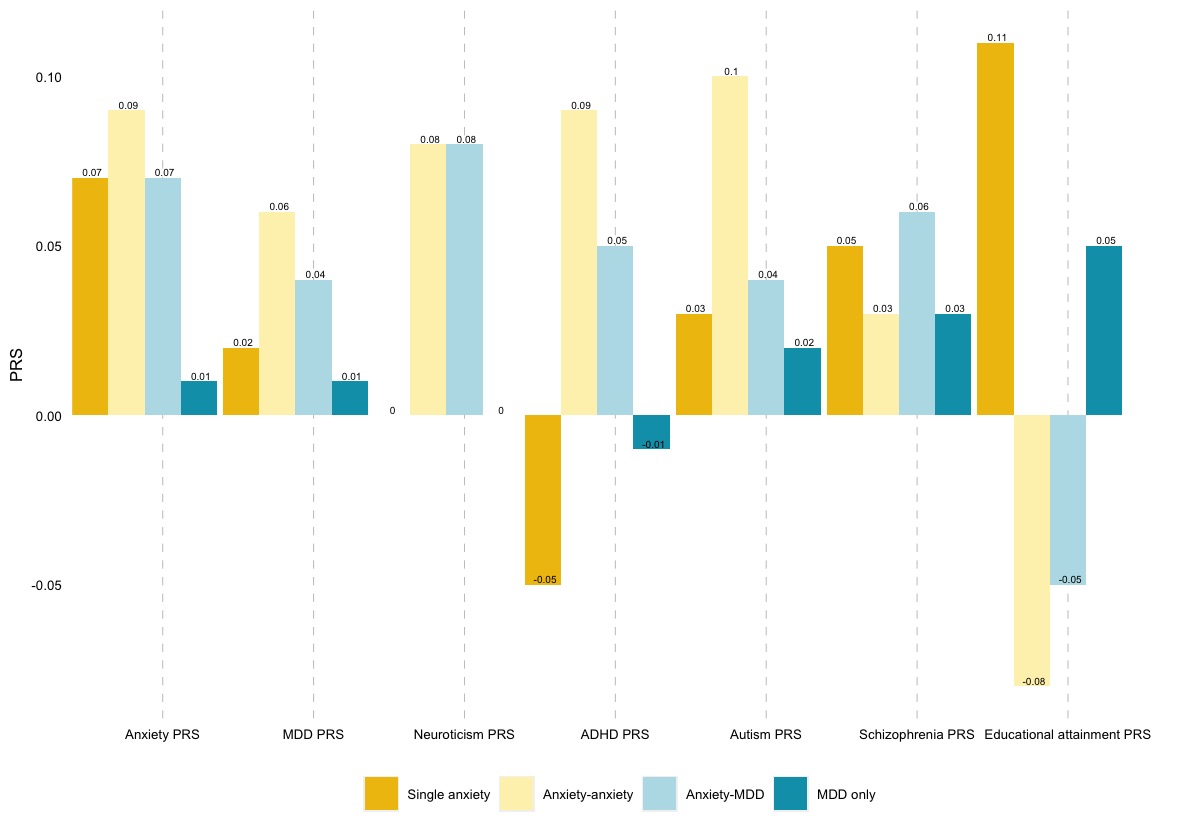


SFigure 7 displays the mean polygenic risk scores for various psychiatric disorders, as specified on the X-axis, by comorbidity group.

*Abbreviations:* PRS = polygenic risk scores. MDD = major depressive disorder. ADHD = attention deficit and hyperactivity disorder.

## SFigure 8. Descriptives for genetic factors by temporal sequence.


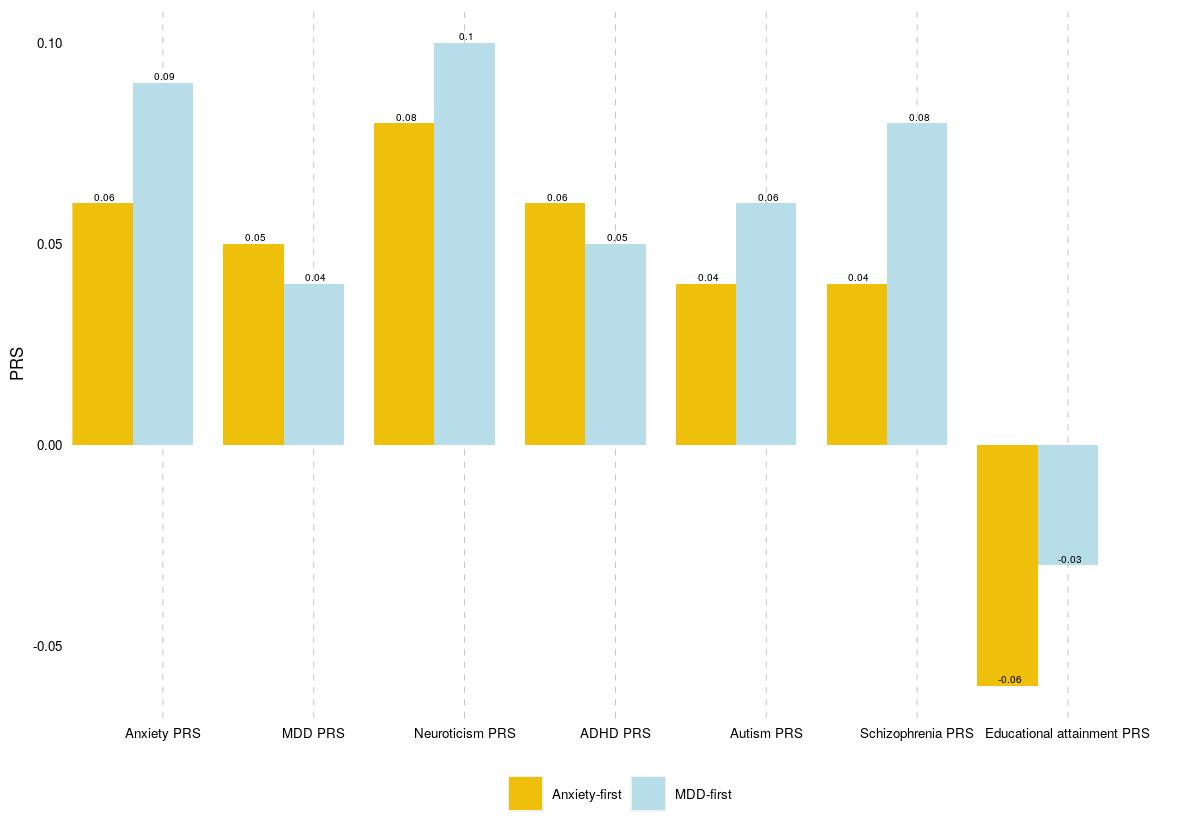
SFigure 8 displays the mean polygenic risk scores for various psychiatric disorders, as specified on the X-axis, by temporal sequence.

*Abbreviations:* PRS = polygenic risk scores. MDD = major depressive disorder. ADHD = attention deficit and hyperactivity disorder.
